# Knockout of integrin αvβ6 protects against renal inflammation in chronic kidney disease by reduction of pro-inflammatory macrophages

**DOI:** 10.1038/s41419-024-06785-5

**Published:** 2024-06-06

**Authors:** Changjian Zhu, Ruilin Zheng, Xu Han, Ziwen Tang, Feng Li, Xinrong Hu, Ruoni Lin, Jiani Shen, Qiaoqiao Pei, Rong Wang, Guangyan Wei, Zhenwei Peng, Wei Chen, Zhou Liang, Yi Zhou

**Affiliations:** 1 Department of Nephrology, The First Affiliated Hospital, Sun Yat-sen University, Guangzhou, 510080 China; 2 NHC Key Laboratory of Clinical Nephrology (Sun Yat-sen University) and Guangdong Provincial Key Laboratory of Nephrology, Guangzhou, 510080 China; 3 Department of Radiation Oncology, The First Affiliated Hospital, Sun Yat-sen University, Guangzhou, 510080 China

**Keywords:** Genetics research, Chronic kidney disease, Chronic inflammation, Cell signalling

## Abstract

Integrin αvβ6 holds promise as a therapeutic target for organ fibrosis, yet targeted therapies are hampered by concerns over inflammatory-related side effects. The role of αvβ6 in renal inflammation remains unknown, and clarifying this issue is crucial for αvβ6-targeted treatment of chronic kidney disease (CKD). Here, we revealed a remarkable positive correlation between overexpressed αvβ6 in proximal tubule cells (PTCs) and renal inflammation in CKD patients and mouse models. Notably, knockout of αvβ6 not only significantly alleviated renal fibrosis but also reduced inflammatory responses in mice, especially the infiltration of pro-inflammatory macrophages. Furthermore, conditional knockout of αvβ6 in PTCs in vivo and co-culture of PTCs with macrophages in vitro showed that depleting αvβ6 in PTCs suppressed the migration and pro-inflammatory differentiation of macrophages. Screening of macrophage activators showed that αvβ6 in PTCs activates macrophages via secreting IL-34. IL-34 produced by PTCs was significantly diminished by αvβ6 silencing, and reintroduction of IL-34 restored macrophage activities, while anti-IL-34 antibody restrained macrophage activities enhanced by αvβ6 overexpression. Moreover, RNA-sequencing of PTCs and verification experiments demonstrated that silencing αvβ6 in PTCs blocked hypoxia-stimulated IL-34 upregulation and secretion by inhibiting YAP expression, dephosphorylation, and nuclear translocation, which resulted in the activation of Hippo signaling. While application of a YAP agonist effectively recurred IL-34 production by PTCs, enhancing the subsequent macrophage migration and activation. Besides, reduced IL-34 expression and YAP activation were also observed in global or PTCs-specific αvβ6-deficient injured kidneys. Collectively, our research elucidates the pro-inflammatory function and YAP/IL-34/macrophage axis-mediated mechanism of αvβ6 in renal inflammation, providing a solid rationale for the use of αvβ6 inhibition to treat kidney inflammation and fibrosis.

## Introduction

Chronic kidney disease (CKD) is a global public health threat with high morbidity and mortality, affecting about 9.1-13.4% of the general population and causing over a million deaths annually worldwide [1]. Renal fibrosis is a common and dynamic pathological process that drives nearly all types of kidney dysfunction to progress to CKD, eventually resulting in renal failure [2]. However, current therapies for CKD primarily address symptoms rather than directly ameliorating kidney fibrosis [3]. Therefore, it is urgent to develop safe and effective treatments to halt this life-threatening process.

Integrin αvβ6 is a member of the integrin family, a group of transmembrane receptors that play crucial roles in cell adhesion and communication between cells and their surrounding extracellular matrix. Previous research showed that integrin αvβ6 is up-regulated during multiple organ fibrosis, e.g., lung, liver, and kidney, and promotes fibrosis via activating the key profibrotic mediator, transforming growth factor-β1 (TGF-β1). This has positioned it as a promising therapeutic target for organ fibrosis [4–8]. Recently, the inhibitor of αvβ6 has progressed to a clinical trial for idiopathic pulmonary fibrosis (IPF) [9]. However, this clinical trial suffered a setback due to severe, unforeseen safety concerns, as IPF patients receiving αvβ6 inhibitor experienced pulmonary inflammation-related complications, including pneumonia, bronchitis, and acute IPF exacerbation [10]. These adverse events are suspected to stem from the pro-inflammatory potential of TGF-β inhibition, highlighting inflammation as the major concern in αvβ6 interventions. However, the current understanding of αvβ6’s involvement in renal inflammation is not well-defined, which impedes further clinical advancement of this target in renal fibrosis. Hence, we aimed to uncover the exact role and mechanism of αvβ6 in renal inflammation during kidney fibrosis.

In the present study, by analyzing public sequencing datasets and experimental verification with kidney samples from CKD patients and murine models, we found that integrin αvβ6 was overexpressed in the proximal tubule cells (PTCs) after kidney injuries and throughout fibrogenesis, positively associating with the exacerbation of renal inflammation. Notably, knockout of αvβ6 in mice remarkably reduced pro-inflammatory macrophage infiltration and inflammation-related mediators, as well as subsequent kidney fibrosis. Further unbiased transcript sequencing and molecular analysis revealed that αvβ6 promoted tubular IL-34 production via activating the Hippo/YAP signaling pathway, which in turn potentiated macrophage infiltration and pro-inflammatory differentiation, leading to renal inflammation and fibrosis. Collectively, our data clarifies the function and detailed mechanism of αvβ6 in renal inflammation, endorsing the use of readily available αvβ6 inhibitors to down-regulate inflammation and fibrosis in CKD patients.

## Results

### Increased integrin αvβ6 in human CKD kidneys is positively associated with inflammatory mediators and immune infiltration

**Fig. 1:**
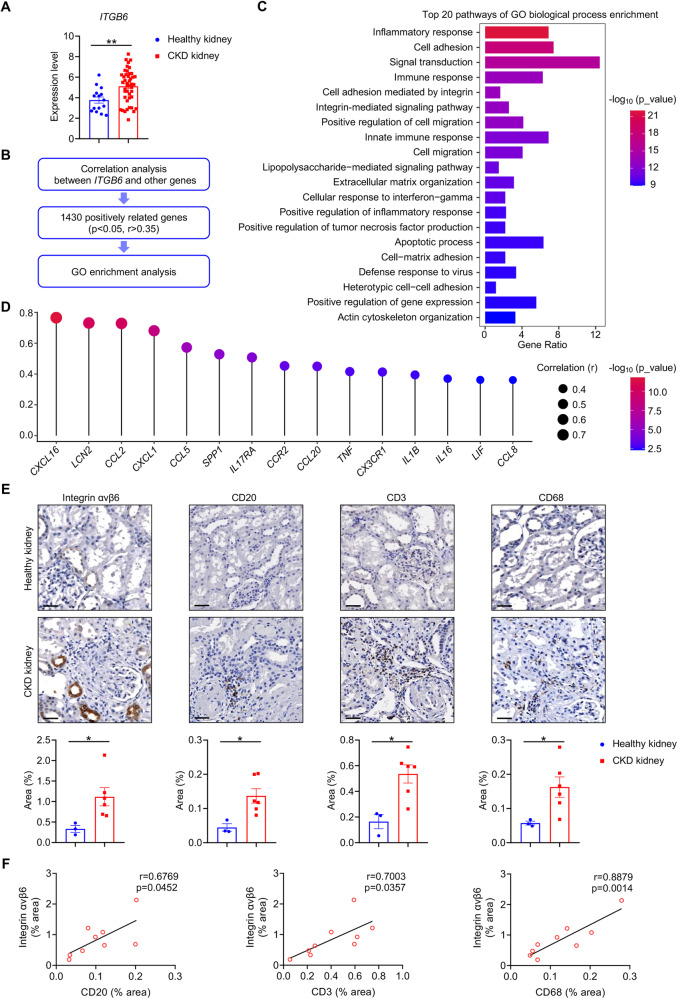
Increased integrin αvβ6 in human CKD kidneys is positively associated with inflammatory mediators and immune infiltration. **A** The expression levels of *ITGB6* in kidney specimens from healthy controls (*n* = 15) and CKD patients (*n* = 59) of dataset GSE180394 were compared. **B** A flow chart of correlation research between *ITGB6* and other genes. **C** GO functional enrichment analysis of the genes positively associated with *ITGB6*. GO, gene ontology. **D** The correlations between the transcription expression level of *ITGB6* and inflammatory mediators in kidney specimens of dataset GSE180394 were analyzed and visualized with lollipop graph. **E** Representative immunostaining images and comparisions of integrin αvβ6, CD20 (a marker of B cells), CD3 (a marker of T cells), and CD68 (a marker of macrophages) in human kidney sections from healthy controls (*n* = 3) and CKD patients (*n* = 6) (scale bar, 50 μm). **F** Correlations between integrin αvβ6 staining quantification and CD3, CD20, and CD68 staining quantifications were analyzed (*n* = 9). Data are presented as mean ± SEM of three biological replicates. Student’s *t* test (**A**, **E**), and Pearson correlation test (**D**, **F**) were performed. *p < 0.05; **p < 0.01.

To explore the relationship between inflammation and integrin αvβ6 in kidney fibrosis, we initiated our study with a bioinformatic analysis of public RNA profiles from CKD patients. According to the inclusion criteria detailed in the methods, we selected the dataset GSE180394 [11], which contains kidney biopsies from healthy individuals and CKD patients with different etiologies, including diabetic nephropathy (DN), lupus nephritis (LN), hypertensive nephropathy (HN), IgA nephropathy (IgAN), focal segmental glomerulosclerosis (FSGS), membranous nephropathy (MN), and minimal change disease (MCD). Our finding from this dataset showed that *ITGB6*, the gene encoding integrin αvβ6, was elevated in the kidneys of CKD patients compared to healthy controls (Fig. 1A). Subsequently, a comprehensive correlation analysis between *ITGB6* and other genes in this dataset was performed. With the standard of *p* < 0.05 and correlation coefficient (r) > 0.35 [12, 13], 1430 positively correlated genes were screened out and then subjected to Gene Ontology (GO) functional analysis to decipher their biological functions (Fig. 1B). Interestingly, in addition to the typical functions of integrin αvβ6, such as cell adhesion, extracellular matrix organization, and cell-matrix adhesion, these *ITGB6*-correlated genes were significantly enriched in inflammation-related biological processes, such as inflammatory response, immune response, innate immune response, and positive regulation of inflammatory response (Fig. 1C). Moreover, positive correlations between *ITGB6* expression and inflammatory mediators, known to contribute to renal inflammation during fibrosis, were also observed, including *CXCL16*, *LCN2*, *CCL2*, *CXCL1*, *CCL5*, *SPP1*, *IL17RA*, *CCR2*, *CCL20*, *TNF*, *CX3CR1*, *IL1B*, *IL16*, *LIF*, and *CCL8* (Fig. 1D and Fig. S1) [14–27].

Immune cells are critical in organ inflammation and are indicative of the kidney inflammatory state [28–30]. We next examined the association between integrin αvβ6 and inflammatory cell infiltration with immunocytochemistry staining in kidney biopsies from healthy controls and CKD patients (Table S1). Compared to normal kidneys, expression of αvβ6 and accumulation of CD20 positive B cells, CD3 positive T cells, and CD68 positive macrophages were markedly increased in CKD patient kidneys (Fig. 1E). Moreover, αvβ6 levels exhibited positive correlations with these immune cells (Fig. 1F).

To sum up, these bioinformatic and experimental results indicate that the elevation of integrin αvβ6 is likely involved in the aggravation of renal inflammation in CKD patients.

### Integrin αvβ6 deficiency reduces renal inflammation and pro-inflammatory macrophage infiltration in injured kidneys

**Fig. 2:**
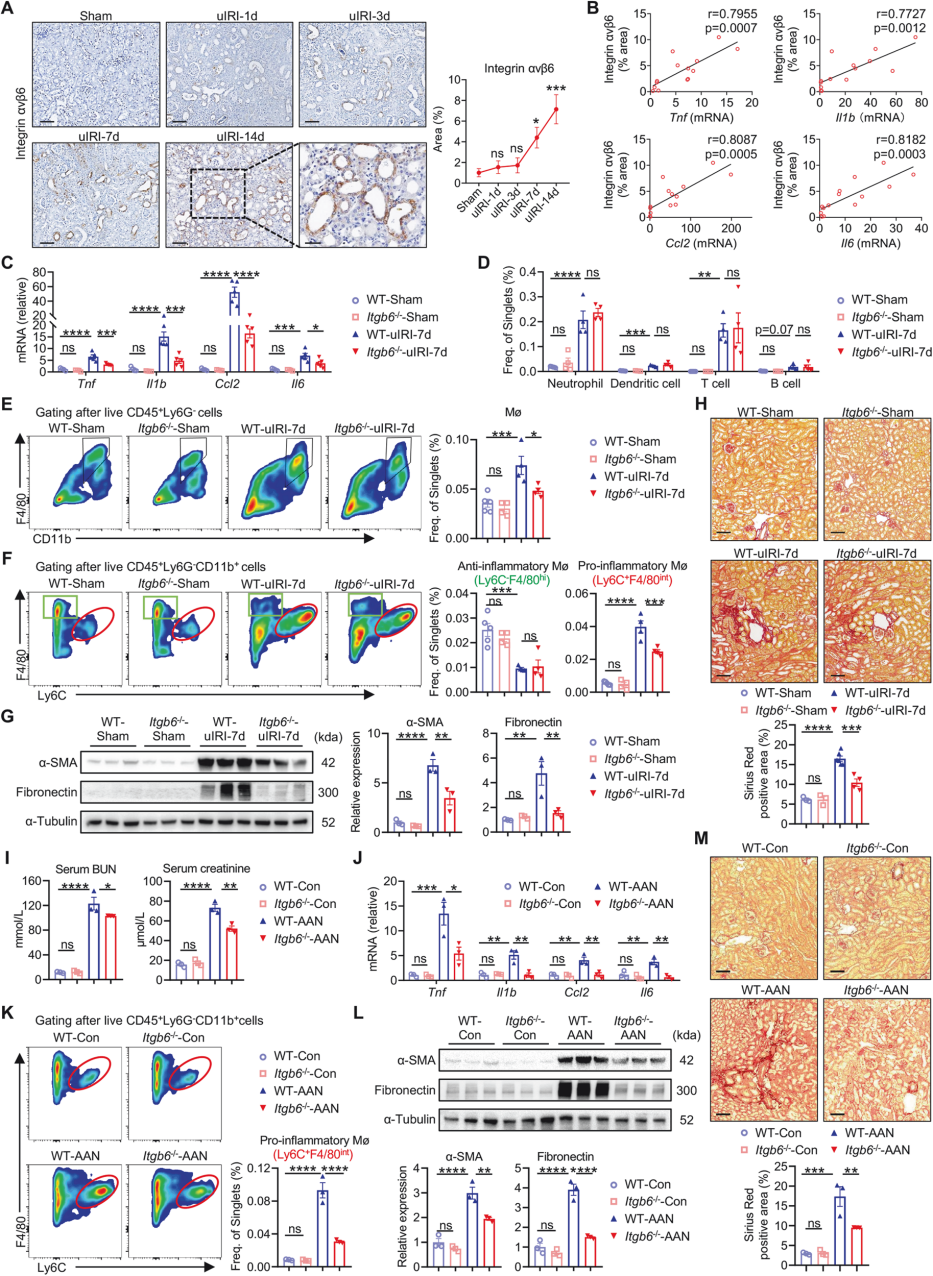
Integrin αvβ6 deficiency reduces renal inflammation and pro-inflammatory macrophage infiltration in injured kidneys. **A** Representative immunostaining images and quantification of integrin αvβ6 in kidney sections from sham and uIRI mice on days 1, 3, 7, and 14 (scale bar, 100 μm). The boxed area in the uIRI-14d panel is magnified in the right panel (scale bar, 50 μm). (*n* = 4-5 per group). **B** Correlations between integrin αvβ6 staining quantification and renal inflammation of sham, uIRI-7d, and uIRI-14d mice. For renal inflammation, see Fig. S2A, it is indicated by *Tnf*, *Il1b*, *Ccl2*, and *Il6* relative mRNA levels detected by qPCR (*n* = 14). **C** Relative mRNA levels of renal inflammatory factors (*Tnf*, *Il1b*, *Ccl2*, and *Il6*) in WT and *Itgb6*^-/-^ mice were detected by qPCR (*n* = 3–5 per group). **D**, **E** Neutrophils, dendritic cells, T cells, B cells, and macrophages in kidneys of sham or uIRI-7d mice were detected by flow cytometry and compared. The gating strategies and representative images of different immune cells are shown in Fig. S3A, B (*n* = 3–5 per group). **F** Comparisons of anti-inflammatory (Ly6c^-^F4/80^hi^) or pro-inflammatory (Ly6c^+^F4/80^int^) macrophages in kidneys of sham or uIRI-7d mice (*n* = 3–5 per group). **G** Western blot of α-SMA and fibronectin in sham or uIRI-7d kidneys. The quantifications of the relative levels of α-SMA/α-Tubulin and fibronectin/α-Tubulin are shown (*n* = 3 per group). **H** Representative images and quantifications of Sirius Red staining in kidney sections from sham or uIRI-7d mice (scale bar, 100 μm) (*n* = 3–6 per group). **I** Serum BUN and creatinine in control or AAN mice. (*n* = 3 per group). **J** Relative mRNA levels of renal inflammatory factors (*Tnf*, *Il1b*, *Ccl2*, and *Il6*) in control or AAN mice were detected by qPCR (*n* = 3 per group). **K** Comparison of pro-inflammatory (Ly6c^+^F4/80^int^) macrophages in control or AAN mice kidneys (*n* = 3 per group). **L** Western blot of α-SMA and fibronectin in control or AAN mice kidneys. The quantifications of the relative levels of α-SMA/α-Tubulin and fibronectin/α-Tubulin are shown (*n* = 3 per group). **M** Representative images and quantifications of Sirius Red staining in kidney sections from control or AAN mice (scale bar, 100 μm) (*n* = 3 per group). Data are presented as mean ± SEM of three biological replicates. One-way ANOVA (**A**, **C**–**M**), and Pearson correlation test (**B**) were performed. *p < 0.05; **p < 0.01; ***p < 0.001; ****p < 0.0001; ns, not significant.

To further characterize αvβ6 in renal inflammation, a unilateral renal ischemia-reperfusion injury (uIRI)-induced renal fibrosis murine model was adopted. Integrin αvβ6 was not obviously altered in the early phase after uIRI injury (day 1, 3) but was remarkably increased in the late phase (day 7, 14) (Fig. 2A). Therefore, we detected the inflammatory and fibrotic phenotypes in uIRI-7d and uIRI-14d mice, and identified severe inflammation and fibrosis in these damaged kidneys, indicated by the significantly increased inflammatory factors (*Tnf*, *Il1b*, *Ccl2*, and *Il6*), enhanced macrophage infiltration, and enlarged fibrotic area (Fig. S2A–C). Further correlation analysis showed that αvβ6 expression was positively associated with kidney inflammation in sham, uIRI-7d, and uIRI-14d mice (Fig. 2B). Consequently, we selected uIRI-7d for subsequent studies.

We then established αvβ6 knockout (*Itgb6*^-/-^)-uIRI mice to determine the exact role of αvβ6 in renal inflammation and renal immune infiltration (Fig. S2D). There was no difference between wild-type (WT)- and *Itgb6*^-/-^-sham groups, but *Itgb6*^-/-^-uIRI-7d mice exhibited a significant reduction in mRNA expression levels of inflammatory indicators, such as *Tnf*, *Il1b*, *Ccl2*, and *Il6* compared to WT-uIRI counterparts (Fig. 2C), indicating that integrin αvβ6 was able to modulate renal inflammation induced by kidney injury. Besides, flow cytometry analysis revealed an increase in all detected immune cells in the injured kidneys. However, only the proportion of macrophages was reduced after *Itgb6* deletion, while other immune cells like neutrophils, dendritic cells, T cells, and B cells, remained unchanged (Fig. 2D, E and Fig. S3A, B). Macrophages were further classified into Ly6C^+^F4/80^int^ infiltrating pro-inflammatory subset and Ly6C^-^F4/80^hi^ resident anti-inflammatory subset [17, 31–33]. The former was up-regulated in uIRI kidneys, while the latter was down-regulated compared with sham control (Fig. 2F). Remarkably, the proportion of pro-inflammatory macrophages was approximately halved in *Itgb6*^-/-^-uIRI-7d mice, whereas the level of the anti-inflammatory subset remained unaffected (Fig. 2F), highlighting that αvβ6 predominantly regulates renal infiltration of inflammatory macrophages. Along with the reduction of inflammation, renal fibrosis in *Itgb6*^-/-^-uIRI-7d mice was significantly alleviated, manifested by lower expression of α-SMA and fibronectin as well as diminished Sirius red-stained area (Fig. 2G, H).

We then validated these findings in the aristolochic acid (AA) injection-induced nephropathy (AAN) mice, a renal fibrosis model with impaired renal function (Fig. 2I). We found that integrin αvβ6 was up-regulated in AAN mice kidneys as well (Fig. S2E), and *Itgb6*^-/-^-AAN mice kidneys also showed decreased renal inflammation, pro-inflammatory macrophages, and fibrosis compared to WT-AAN mice (Fig. 2J–M). Notably, αvβ6 knockout led to improved renal function in AAN mice, as evidenced by reduced blood urea nitrogen (BUN) and serum creatinine (Fig. 2I). Collectively, these results demonstrated that knockout of integrin αvβ6 attenuated renal inflammation during fibrotic progression, primarily by reducing inflammatory macrophage infiltration.

### Integrin αvβ6 knockdown on PTCs effectively blocks macrophage migration and proinflammatory differentiation

**Fig. 3:**
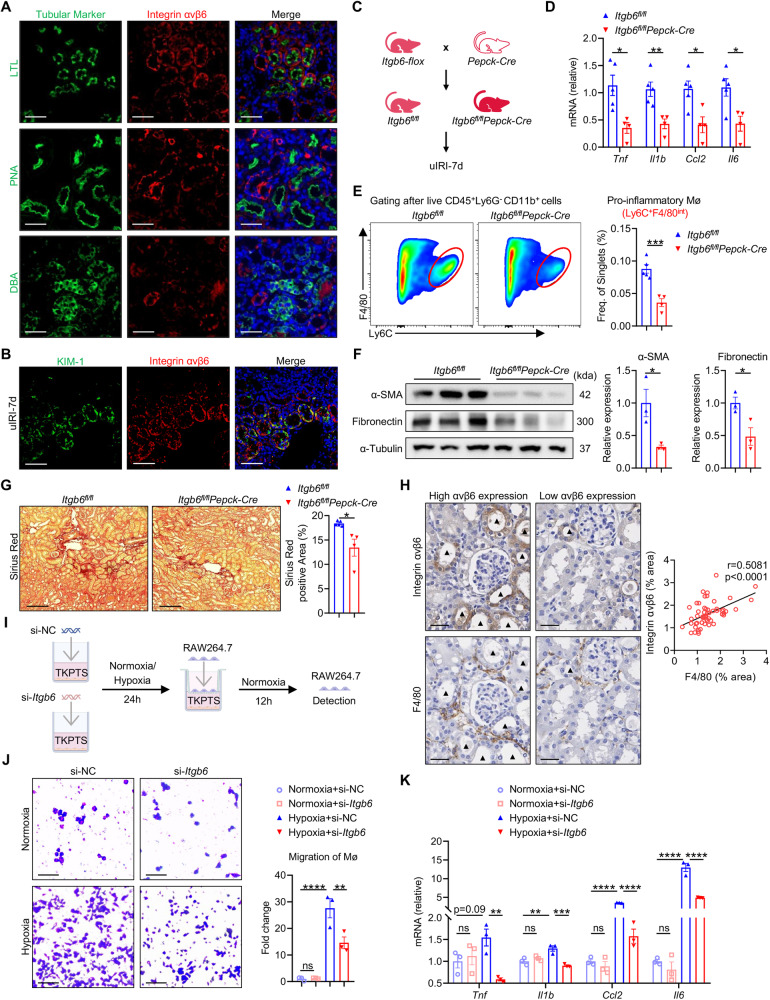
Integrin αvβ6 knockdown on PTCs effectively blocks macrophage migration and proinflammatory differentiation. **A** Immunofluorescence staining for different segment-specific tubular markers (green), integrin αvβ6 (red), and DAPI (blue) in WT-uIRI-7d kidney sections (scale bar, 50 μm). Segment-specific tubular markers were used as follows: proximal tubule, LTL; distal tubule, PNA; and collecting duct, DBA. **B** Immunofluorescence staining for KIM-1 (green), integrin αvβ6 (red), and DAPI (blue) in uIRI-7d kidney sections (scale bar, 50 μm). **C** Schematic of generating *Itgb6*^*fl/fl*^*Pepck-Cre* or *Itgb6*^*fl/fl*^ mice which delete αvβ6 specific in PTCs or not and experimental design. **D** Relative mRNA levels of renal inflammatory factors (*Tnf*, *Il1b*, *Ccl2*, and *Il6*) in *Itgb6*^*fl/fl*^*Pepck-Cre*- or *Itgb6*^*fl/fl*^-uIRI-7d mice were detected by qPCR (*n* = 4-5 per group). **E** Comparison of pro-inflammatory (Ly6c^+^F4/80^int^) macrophages in *Itgb6*^*fl/fl*^*Pepck-Cre*- or *Itgb6*^*fl/fl*^-uIRI-7d mice kidneys (*n* = 4-5 per group). **F** Western blot of α-SMA and fibronectin in *Itgb6*^*fl/fl*^*Pepck-Cre*- or *Itgb6*^*fl/fl*^-uIRI-7d mice kidneys. The quantifications of the relative levels of α-SMA/α-Tubulin and fibronectin/α-Tubulin are shown (*n* = 4-5 per group). **G** Representative images and quantifications of Sirius Red staining in kidney sections from *Itgb6*^*fl/fl*^*Pepck-Cre*- or *Itgb6*^*fl/fl*^-uIRI-7d mice (scale bar, 100 μm) (*n* = 4-5 per group). **H** Representative immunostaining images and correlation analysis of integrin αvβ6 and F4/80 in kidney serial sections from WT-uIRI-7d mice (scale bar, 50 μm). Black triangles indicate αvβ6-expressing tubules in serial sections (*n* = 56 fields from 6 mice). **I** Schematic of the in vitro co-culture system. TKPTS cells transfected with si-NC or si-*Itgb6* were pre-stimulated with hypoxia for 24 h or not, and then co-cultured with RAW264.7 cells for 12 h. **J**, **K** Migration and pro-inflammatory differentiation of RAW264.7 cells after co-culturing with si-NC or si-*Itgb6*-transfected TKPTS cells under the hypoxic stimulation or not (scale bar, 25 μm). Pro-inflammatory differentiation is indicated by relative mRNA levels of inflammatory factors (*Tnf*, *Il1b Ccl2*, and *Il6*) (*n* = 3 per group). Data are presented as mean ± SEM of three biological replicates. Student’s *t*-test (**D**–**G**), Pearson correlation test (**H**), and one-way ANOVA (**J**, **K**) were performed. *p < 0.05; **p < 0.01; ***p < 0.001; ****p < 0.0001; ns, not significant.

By analyzing public single-cell RNA sequencing (scRNA-seq) data from uIRI-7d mouse kidneys (GSE139506) [34], we found that *Itgb6* was mainly expressed in tubule cells, particularly within injured proximal tubules (Fig. S4A). To ascertain the precise tubular types αvβ6 expressed on, we performed co-staining of αvβ6 with renal tubular cell markers: lotus tetragonolobus lectin (LTL) for proximal tubules; peanut agglutinin (PNA) for distal convoluted tubules; and dolichos biflorus agglutinin (DBA) for collecting duct epithelium. The results revealed that integrin αvβ6 primarily co-localized with proximal tubular segments, with scant detection in distal tubules and collecting ducts (Fig. 3A). Additional co-staining of αvβ6 with kidney injury molecule 1 (KIM-1) showed that αvβ6 was predominantly expressed on the KIM-1-positive injured tubules (Fig. 3B). These immunostaining results, combined with the scRNA-seq data, demonstrate that αvβ6 was primarily produced by injured PTCs. Therefore, we generated *Itgb6*^*fl/fl*^*Pepck-Cre* mice with PTCs-specific αvβ6 knockout and conducted uIRI (Fig. 3C and Fig. S4B). Compared to *Itgb6*^*fl/fl*^-IRI-7d controls, *Itgb6*^*fl/fl*^*Pepck-Cre*-IRI-7d mice exhibited similar alleviation of kidney inflammation and fibrosis as systemic *Itgb6* knockout mice (Fig. 3D–G). These results emphasized that it is the proximal tubular αvβ6 that promotes renal inflammation and fibrosis after kidney injury.

Considering αvβ6’s role in facilitating macrophage infiltration, as we described above, we then performed immunostaining of αvβ6 and F4/80 with serial kidney sections from uIRI-7d mice to evaluate the spatial relationship between αvβ6-expressing tubules and macrophages. We found that regions with high αvβ6 expression were adjacent with more macrophages than areas with lower expression and αvβ6 expression levels were positively correlated with the presence of macrophages (Fig. 3H), indicating that αvβ6 expression by injured proximal tubules is closely related to macrophage infiltration after kidney damage.

Next, to validate the role of αvβ6 in the interactions between PTCs and macrophages, we established an in vitro transwell co-culture system. PTCs (TKPTS) transfected with si-RNA (si-NC or si-*Itgb6*) were hypoxically stimulated for 24 h or not, and then co-cultured with macrophages (RAW264.7) under normoxic conditions for 12 h (Fig. 3I). Hypoxia was shown to up-regulate αvβ6 expression in PTCs, while si-*Itgb6* effectively down-regulated it (Fig. S4C, D). Additionally, under hypoxic conditions, PTCs markedly attracted macrophages to migrate in the transwell chambers, and *Itgb6* silencing alleviated this response (Fig. 3J). Moreover, si-*Itgb6* transfection suppressed the pro-inflammatory activation of macrophages induced by hypoxic PTCs, represented by the decreased expression levels of *Tnf*, *Il1b*, *Ccl2*, and *Il6* (Fig. 3K).

Altogether, we can conclude that proximal tubular αvβ6 can promote the migratory and pro-inflammatory abilities of macrophages to exacerbate renal inflammation and fibrosis.

### Proximal tubular integrin αvβ6 facilitates macrophage migration and activation through upregulation and secretion of IL-34

**Fig. 4:**
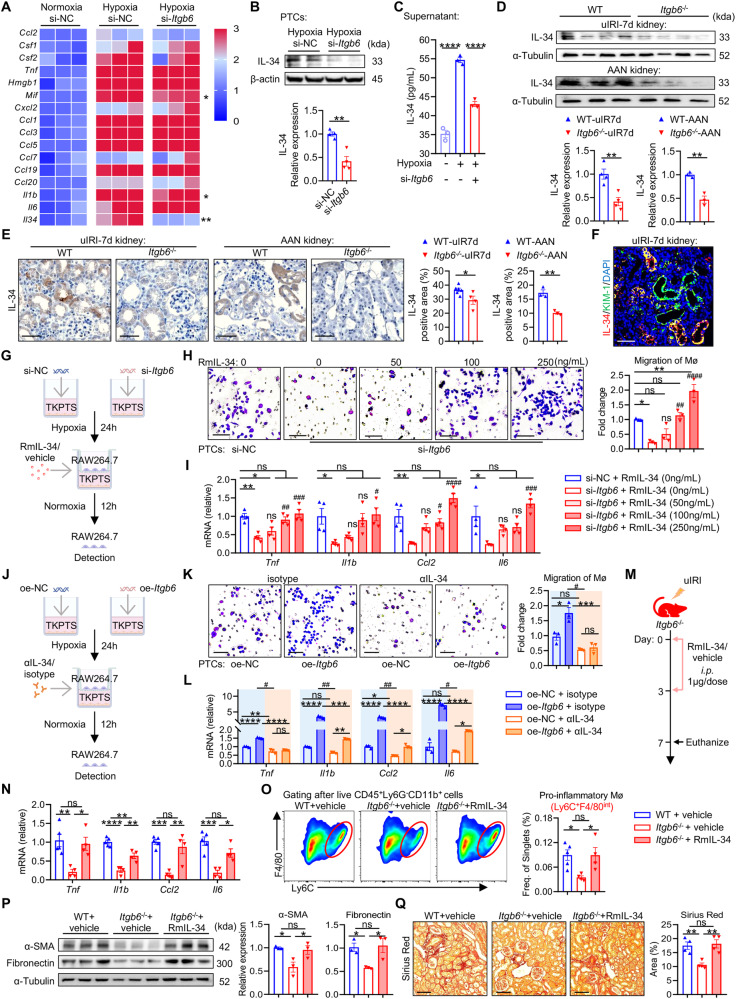
Proximal tubular integrin αvβ6 facilitates macrophage activities through upregulation and secretion of IL-34. **A** TKPTS cells transfected with si-NC or si-*Itgb6* were pre-stimulated with hypoxia for 24 h or not, and then the relative mRNA levels of macrophage activators in TKPTS cells were detected with qPCR and shown with heatmap (*n* = 3 per group). **B** Western blot of IL-34 in si-NC- or si-*Itgb6*-transfected hypoxic TKPTS cells. The quantification of the relative levels of IL-34/β-actin is shown (*n* = 4 per group). **C** TKPTS cells transfected with si-NC or si-*Itgb6* were pre-stimulated with hypoxia for 24 h or not, and then IL-34 in supernatant was detected by ELISA (*n* = 3 per group). **D**, **E** Western blot and immunostaining of IL-34 in uIRI-7d or AAN kidneys from WT and *Itgb6*^-/-^ mice (scale bar, 50 μm). The quantification of the relative levels of IL-34/α-Tubulin and quantification of IL-34 positive area proportion are shown (*n* = 3–5 per group). **F** Immunofluorescence staining for IL-34 (red), KIM-1 (green), and DAPI (blue) in WT-uIRI-7d kidney sections (scale bar, 50 μm). **G** Schematic of an in vitro rescue experiment. TKPTS cells transfected with si-NC or si-*Itgb6* were pre-stimulated with hypoxia for 24 h or not, and then co-cultured with RAW264.7 cells for 12 h. RmIL-34 or vehicle was supplemented into the co-culture system as indicated. **H**, **I** Migration and pro-inflammatory differentiation of RAW264.7 cells in the co-culture system (scale bar, 25 μm) (*n* = 3-4 per group). **J** Schematic of another in vitro reverse rescue experiment. TKPTS cells transfected with oe-NC or oe-*Itgb6* plasmids were pre-stimulated with hypoxia for 24 h or not, and then co-cultured with RAW264.7 cells for 12 h. Anti-IL-34 antibody (αIL-34) or isotype control (10 μg/mL) was added into the co-culture system as indicated. **K**, **L** Migration and pro-inflammatory differentiation of RAW264.7 cells in the co-culture system (scale bar, 25 μm) (*n* = 3 per group). **M** Schematic of an in vivo rescue experiment. *Itgb6*^-/-^ mice were intraperitoneally supplemented with recombinant mouse IL-34 (rmIL-34) or vehicle (1 μg/dose) immediately and 3 days after uIRI, and WT-IRI-7d mice received vehicle served as controls. **N** Relative mRNA levels of renal inflammatory factors (*Tnα*, *Il1b*, *Ccl2*, and *Il6*) in WT and *Itgb6*^-/-^ mice supplemented with rmIL-34 or vehicle were detected by qPCR (*n* = 4-5 per group). **O** Comparison of pro-inflammatory (Ly6c^+^F4/80^int^) macrophages in WT and *Itgb6*^-/-^ mice supplemented with rmIL-34 or vehicle (*n* = 4-5 per group). **P** Western blot of α-SMA and fibronectin in kidneys of WT and *Itgb6*^-/-^ mice supplemented with rmIL-34 or vehicle. The quantifications of the relative levels of α-SMA/α-Tubulin and fibronectin/α-Tubulin are shown (*n* = 3 per group). **Q** Representative images and quantifications of Sirius Red staining in kidney sections from WT and *Itgb6*^-/-^ mice supplemented with rmIL-34 or vehicle (scale bar, 100 μm) (*n* = 4-5 per group). Data are presented as mean ± SEM of three biological replicates. Student’s *t*-test (**B**, **D**, **E**, **K**, **L**) and one-way ANOVA (**C**, **H**–**Q**) were performed. *p < 0.05; **p < 0.01; ***p < 0.001; ****p < 0.0001. ^#^*p* < 0.05; ^##^*p* < 0.01; ^###^*p* < 0.001; ^####^*p* < 0.0001; compared with si-*Itgb6* + rmIL-34 group (0 ng/mL) (**H**, **I**); ^#^*p* < 0.05; ^##^*p* < 0.01; up-regulated degree by oe-*Itgb6* was compared between isotype (light blue background) and αIL-34 group (light orange background) (**K**, **L**); ns, not significant.

Recognizing that αvβ6 is mainly located on the cell membrane but not known to be secreted [35], we hypothesized the existence of an αvβ6-controlled mediator facilitates communication between these two cells. Thus, we performed a comprehensive qPCR screening for common activators of macrophage in tubular cells, including *Ccl2*, *Csf1*, *Csf2*, *Tnf*, *Hmgb1*, *Mif*, *Cxcl2*, *Ccl1*, *Ccl3*, *Ccl5*, *Ccl7*, *Ccl19*, *Ccl20*, *Il1b*, *Il6*, and *Il34* [36–51]. Following hypoxia stimulation, most of these mediators in PTCs were strongly up-regulated; however, *Itgb6* deletion only inhibited the increase of *Mif*, *Il1b*, and *Il34*, with *Il34* showing the most prominent reduction (Fig. 4A). These changes of IL-34 were confirmed by western blot (Fig. 4B). We further examined secreted IL-34 levels in tubular culture supernatants and observed similar alterations (Fig. 4C), indicating that secreted IL-34 regulated by tubular αvβ6 may serve as an intermediary between PTCs and macrophages.

Modulation of IL-34 was also detected in mouse kidneys. *Itgb6* knockout had no impact on renal IL-34 expression in the sham group (Fig. S5A, B), but obviously lessened the upregulation of IL-34 in uIRI-7d and AAN kidneys (Fig. 4D and Fig. S5C, D). Immunostaining displayed less renal tubular IL-34 in *Itgb6*^-/-^-uIRI/AAN mice than WT-uIRI/AAN mice as well (Fig. 4E). Notably, similar to the expression pattern of αvβ6, IL-34 was primarily expressed in injured proximal tubules [34] (Fig. S5H and Fig. 4F), and conditionally knocking out αvβ6 in PTCs resulted in a comparable reduction of tubular IL-34 (Fig. S5E–G). These results indicate that IL-34 is a downstream cytokine regulated by αvβ6 in PTCs.

We then conducted in vitro experiments to confirm IL-34’s role in mediating tubular αvβ6-regulated macrophage activation. Firstly, we set up a rescue experiment where recombinant mouse IL-34 (rmIL-34) was supplemented to the co-culture system of si-*Itgb6*-transfected TKPTS and RAW264.7 cells (Fig. 4G). We found that the addition of rmIL-34 restored the migration and pro-inflammatory differentiation of macrophages inhibited by si-*Itgb6*-transfected PTCs, in a concentration-dependent manner (Fig. 4H, I). In addition, TKPTS cells with or without overexpression of αvβ6 (oe-*Itgb6* or oe-NC) (Fig. S5I) were co-cultured with macrophages in the presence of an anti-IL-34 antibody (αIL-34) or isotype control (Fig. 4J). Under hypoxic conditions, overexpression of αvβ6 in PTCs markedly enhanced the migration of macrophages and their production of inflammatory mediators. However, these upregulation effects by αvβ6 overexpression were effectively inhibited after αIL-34 application (Fig. 4K, L).

Finally, we validated IL-34’s role in vivo by supplementing rmIL-34 to *Itgb6*^-/-^-IRI-7d mice (Fig. 4M). The addition of rmIL-34 effectively restored the levels of renal inflammatory factors, pro-inflammatory macrophage infiltration, and the extent of subsequent fibrosis in *Itgb6*^-/-^-IRI-7d mice, to those seen in WT-IRI-7d (Fig. 4N–Q). Consequently, these data demonstrate that IL-34 mediated αvβ6-regulated renal inflammation and fibrosis after kidney injury. A recent study [52] has reported that IL-34 may up-regulate the polarization of M2 macrophage, a critical contributor to renal fibrosis [28], after renal transplantation. Similarly, here, we observed that the proportion of renal M2 macrophage in *Itgb6*^-/-^-uIRI mice was obviously decreased compared to WT-uIRI mice, and rmIL-34 supplementation tended to restore the level of M2 macrophage (Fig. S5J), indicating that integrin αvβ6 may also influence M2 macrophage polarization via upregulation of IL-34, thereby potentially contributing to renal fibrosis.

### Loss of integrin αvβ6 suppresses the activation of tubular Hippo/YAP signaling pathway

**Fig. 5:**
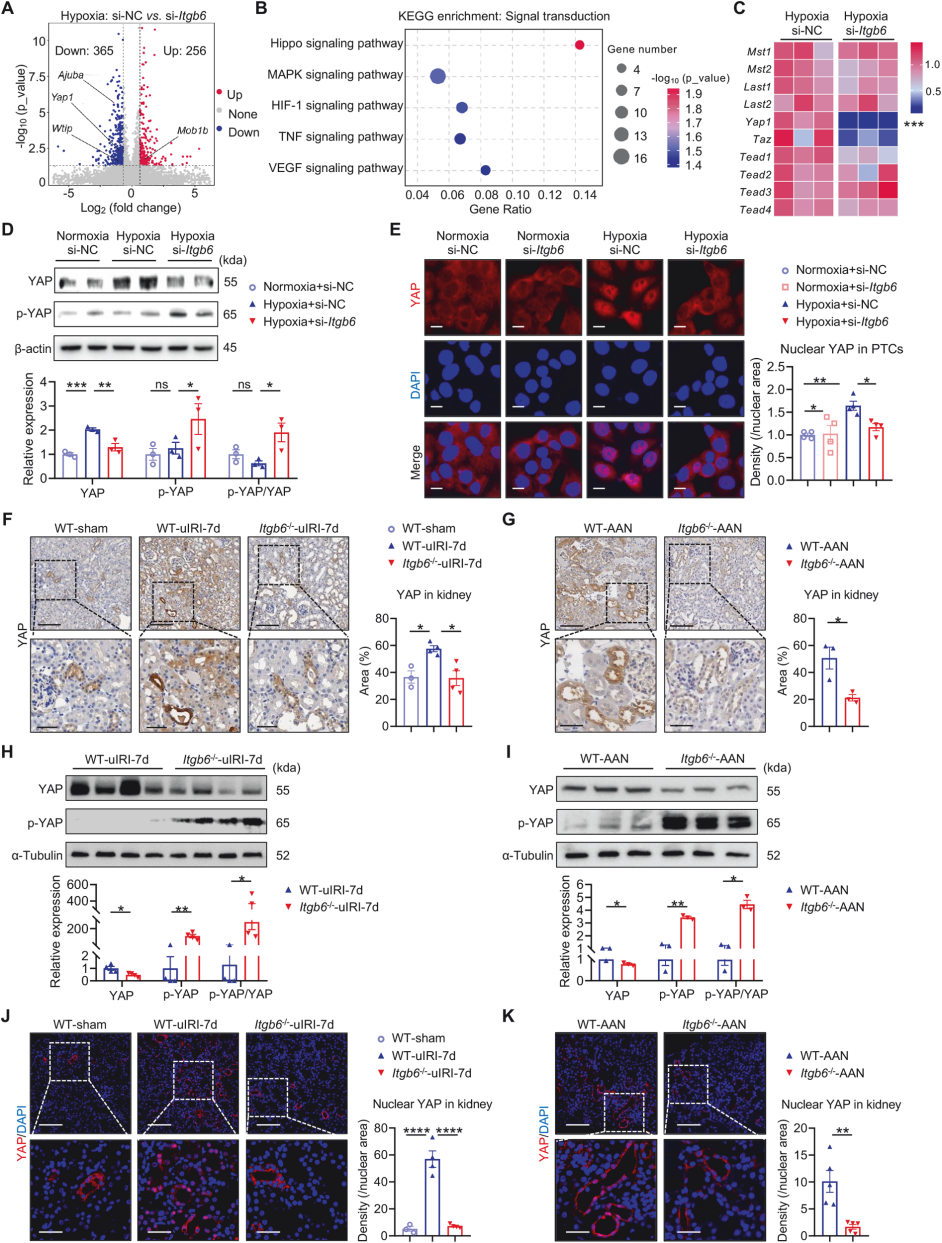
Loss of integrin αvβ6 suppresses the activation of tubular Hippo/YAP signaling pathway. **A** DEGs obtained from the comparisons between si-NC and si-*Itgb6*-transfected hypoxic TKPTS cells in RNA-sequencing. **B** KEGG enrichment analysis of DEGs in RNA-sequencing. **C** Relative mRNA levels of the important molecules in the Hippo/YAP pathway in si-NC or si-*Itgb6*-transfected hypoxic TKPTS cells were detected by qPCR (*n* = 3 per group). **D** Western blot of total YAP and Ser127-phosphorylated YAP (p-YAP) in si-NC or si-*Itgb6*-transfected hypoxic TKPTS cells. The quantifications of the relative levels of t-YAP/β-actin, p-YAP/β-actin, and p-YAP/YAP are shown (*n* = 3 per group). **E** Representative images of immunofluorescence staining for YAP (red) and DAPI (blue) in si-NC- or si-*Itgb6*-transfected TKPTS cells under the hypoxic stimulation or not. Semi-quantification of nuclear YAP expression is shown (Scale bar, 10 µm) (*n* = 4 per group). **F**, **G** Immunostaining of YAP in uIRI-7d (**F**) or AAN (**G**) kidneys from WT and *Itgb6*^-/-^ mice. The quantification of YAP positive area proportion is shown (scale bar, 100 μm). The boxed area in the upper panels is magnified in the lower panels (scale bar, 50 μm) (*n* = 4 per group). **H**, **I** Western blot of YAP and p-YAP in uIRI-7d (**H**) or AAN (**I**) kidneys from WT and *Itgb6*^-/-^ mice. The quantifications of the relative levels of YAP/α-Tubulin, p-YAP/α-Tubulin, and p-YAP/YAP are shown (*n* = 3-4 per group). **J**, **K** Representative images of immunofluorescence staining for YAP (red) and DAPI (blue) in uIRI-7d (**J**) or AAN (**K**) kidneys from WT and *Itgb6*^-/-^ mice. Semi-quantification of nuclear YAP expression is shown (Scale bar, 50 µm). The boxed area in the upper panels is magnified in the lower panels (scale bar, 25 μm) (*n* = 3-4 per group). Data are presented as mean ± SEM of three biological replicates. One-way ANOVA (**D**–**F**, **J**), and Student’s *t*-test (**G**–**I**, **K**) were performed. *p < 0.05; **p < 0.01; ***p < 0.001; ****p < 0.0001; ns, not significant.

To delve deeper into the mechanism by which αvβ6 modulates IL-34 expression, an unbiased RNA-sequencing of si-NC and si-*Itgb6*-transfected hypoxic TKPTS cells was performed. Based on previous research [53], we utilized the screening criteria of *p*-value < 0.05 and |fold change| > 1.2, and identified 365 down-regulated and 256 up-regulated differential expressed genes (DEGs) after *Itgb6* silencing, which were visualized with Volcano plot (Fig. 5A). KEGG functional enrichment analysis of these DEGs highlighted the Hippo signaling pathway as significantly enriched in the term of “signal transduction” (Fig. 5B). The Hippo pathway, known for its role in suppressing cell proliferation and regulating tissue homeostasis and organ size, has recently been reported to be implicated in mediating IL-34 expression in breast cancer cells [54]. This led to the hypothesis that αvβ6 might regulate IL-34 expression through the Hippo signaling pathway. Subsequently, a detailed examination of pathway-related genes revealed that yes-associated protein (YAP), a central effector of the Hippo pathway, was significantly down-regulated following *Itgb6* silencing (Fig. 5C). Further western blot confirmed that hypoxia induced a remarkable increase of total YAP expression in TKPTS cells, which was negated in the absence of αvβ6 (Fig. 5D). Therefore, it is reasonable to speculate that αvβ6 might regulate YAP expression thus inhibiting the Hippo pathway, culminating in IL-34 up-expression. In addition, once the Hippo kinase cascade is suppressed, YAP is dephosphorylated, activated, and translocated into the nucleus to initiate transcriptional programs. Therefore, we then detected the phosphorylated YAP (p-YAP) and nuclear translocation of YAP through western blot and immunocytochemistry staining to examine its activation. Notably, p-YAP expression was elevated after silencing *Itgb6* (Fig. 5D). Besides, under normoxic conditions, YAP was predominantly distributed within the cytoplasm of PTCs, and there was no obvious difference between si-NC and si-*Itgb6*-transfected groups. However, hypoxia led to a surge in nuclear YAP, indicative of its translocation from cytoplasm to nucleus. This shift was reversed by si-*Itgb6* treatment, which confined YAP within the cytosol with minimal nuclear presence (Fig. 5E).

Similar results were observed in vivo. Compared to control mice, YAP expression and nuclear translocation in renal tubules of uIRI-7d and AAN mice was significantly increased, and this upregulation was abolished after either global or PTCs-specific deletion of *Itgb6*, whereas p-YAP level was significantly up-regulated in the absence of αvβ6 (Fig. 5F–K, and Fig. S6A–F). In summary, these results suggested that αvβ6 could enhance tubular YAP expression and activate tubular YAP by inhibiting its phosphorylation-mediated degradation and promoting its nuclear translocation.

### YAP activation is essential for the role of integrin αvβ6 in the regulation of IL-34 and macrophages

**Fig. 6:**
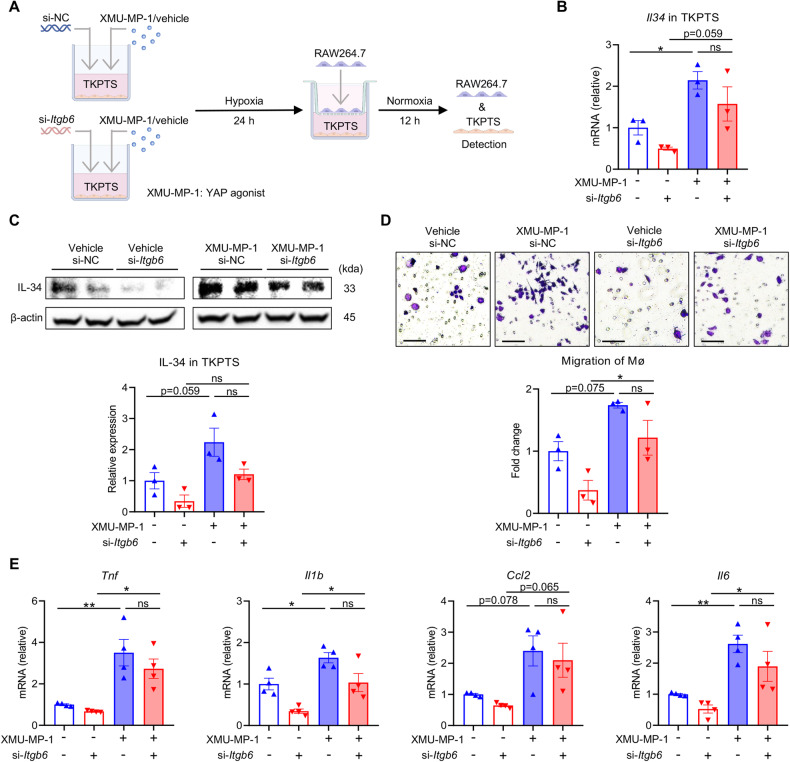
YAP activation is essential for the role of integrin αvβ6 in the regulation of IL-34 and macrophages. **A** The schematic diagram of an in vitro rescue experiment. TKPTS cells transfected with si-NC or si-*Itgb6* were pre-stimulated with hypoxia for 24 h or not, and then co-cultured with RAW264.7 cells for 12 h. XMU-MP-1 (YAP agonist) or vehicle was supplemented into the co-culture system as indicated. **B**, **C** Relative mRNA levels and protein levels of IL-34 in si-NC- or si-*Itgb6*-transfected hypoxic TKPTS cells were determined by qPCR and western blot. The quantification of the relative levels of IL-34/β-actin is shown (**C**) (*n* = 3 per group). **D**, **E** Migration and pro-inflammatory differentiation of RAW264.7 cells in the co-culture system (scale bar, 25 μm) (*n* = 3-4 per group). Data are presented as mean ± SEM of three biological replicates. One-way ANOVA was performed. *p < 0.05; **p < 0.01; ns, not significant.

Since the Hippo/YAP signaling pathway was remarkably regulated by αvβ6, it raised the question of whether it mediates αvβ6’s regulatory effects on IL-34 expression and macrophage infiltration after kidney injury. To explore this, we added XMU-MP-1, a YAP agonist that could activate YAP by suppressing its phosphorylation, into the culture supernatant of hypoxic TKPTS cells (Fig. 6A). Notably, XMU-MP-1 augmented both mRNA and protein levels of IL-34, reversing the reduction of IL-34 induced by *Itgb6* silencing (Fig. 6B, C). Concurrent with IL-34’s alteration, XMU-MP-1 significantly increased the migratory and pro-inflammatory abilities of macrophages and counteracted the suppressive effects induced by si-*Itgb6* transfection (Fig. 6D, E). These results indicated that YAP was a crucial link connecting proximal tubular αvβ6 and IL-34-activated macrophages.

**Fig. 7:**
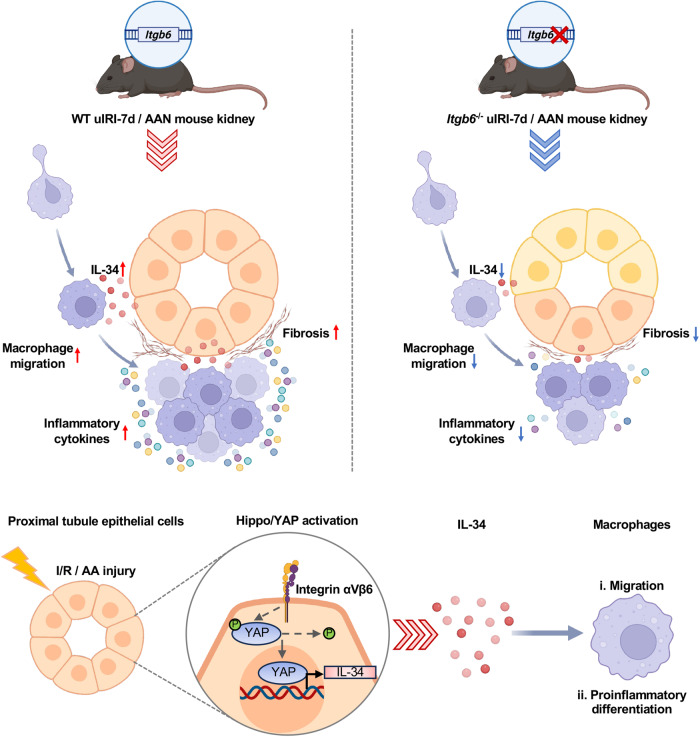
Proposed working model of integrin αvβ6 in the regulation of renal inflammation. After kidney injury, increased αvβ6 in the proximal tubules activates the YAP/IL-34 axis, leading to macrophage infiltration and pro-inflammatory differentiation, ultimately aggravating renal inflammation and fibrosis. Conversely, the absence of αvβ6 diminishes this pathway, thereby alleviating renal inflammation and fibrosis.

Consequently, a working model was proposed based on our research (Fig. 7). Increased proximal tubular αvβ6 after kidney injury activates the YAP/IL-34 axis, leading to macrophage infiltration and pro-inflammatory differentiation, ultimately aggravating renal inflammation and fibrosis. Conversely, the absence of αvβ6 diminishes this pathway, thereby alleviating renal inflammation and fibrosis.

## Discussion

Elevated integrin αvβ6 is crucial in tissue fibrosis, which is acknowledged for its role in inducing aberrant activation of TGF-β, a center player in the fibrotic process [35]. Previous studies have shown that αvβ6 overexpresses in injured kidneys, and knockout of αvβ6 can alleviate kidney fibrosis [6, 55]. Consequently, targeting αvβ6 is emerging as a promising therapeutic strategy for renal fibrosis. Nevertheless, clinical trials targeting αvβ6 in IPF patients were halted due to severe inflammation-related side effects, thought to be associated with TGF-β suppression [10]. While previous studies regarding αvβ6 and kidney disease mainly focused on renal fibrosis, its impact on kidney inflammation remains obscure, which impeded the progress of αvβ6 inhibitors in clinical trials for renal fibrosis. Unraveling the role of αvβ6 in renal inflammation is thus crucial for progressing kidney fibrosis treatments.

Interestingly, αvβ6 appears to regulate tissue inflammation in a complex manner, acting both to promote and inhibit inflammatory pathways. Consistent with the pivotal role in activating TGF-β, deactivating the *Itgb6* gene increases inflammatory cell infiltration in the lungs and skin of mice [56]. Conversely, in some pathological conditions, the application of antibodies against αvβ6 or *Itgb6* knockout suppressed severe inflammatory responses, such as IL-1β-induced infant lung injury and integrin β1 deficiency-induced embryonic skin inflammation [57, 58]. Besides, transgenic αvβ6 overexpression in mice could also promote dextran sodium sulfate (DSS)-induced colitis [59]. These contrasting phenomena indicate that αvβ6 can regulate tissue inflammation in a double-edged way under different conditions, and also emphasize that αvβ6 may have multifaceted roles beyond merely activating TGF-β to suppress inflammation. For renal inflammation, although the involvement of αvβ6 is not fully understood, preliminary researches hint that αvβ6 probably be a potential target for mitigating renal inflammation. Dating back to 1997, researchers have observed that the increased expression of αv integrins is positively associated with macrophage presence in glomerulonephritis [60]. In the murine model of Alport syndrome, αvβ6 inhibition restrained renal pro-inflammatory gene expression [61]. Nevertheless, whether and how αvβ6 modulates inflammation during renal fibrosis requires more solid and direct evidence. Here, by applying both bioinformatic analysis and experimental verification, we observed a significant association between αvβ6 expression and renal inflammation in CKD patients and mouse models. Further functional experiments revealed that αvβ6 knockout effectively attenuated renal inflammation and fibrosis and improved renal function in mice. Our results implied that targeting αvβ6 is a promising strategy for treating renal inflammation and fibrosis, potentially facilitating the recovery of renal function.

We also reported that αvβ6 predominantly regulated the infiltration of pro-inflammatory macrophages. Spatial analysis showed that macrophages tended to be distributed around injured tubules expressing αvβ6. Moreover, both in vivo studies with conditional deletion of αvβ6 in PTCs and in vitro co-culture experiments demonstrated that αvβ6 in PTCs promoted the migration and inflammatory factors production of macrophages. Consistent with our study, previous studies showed that αvβ6 inhibition decreased the infiltration of macrophages in pulmonary and skin inflammation [57, 58]. Nevertheless, the underlying mechanism is still unknown. In the present study, through screening the crucial activator of macrophages, we identified IL-34 as the key mediator influenced by αvβ6. Subsequent in vitro and in vivo experiments verified that αvβ6 could regulate tubular IL-34 expression. Notably, the application of rmIL-34 restored macrophage migration and pro-inflammatory differentiation, effectively reversing the effects of αvβ6 deletion. Conversely, IL-34 blockade inhibited the enhanced macrophage activities induced by αvβ6 overexpression in PTCs. Additionally, we also observed that IL-34, up-regulated by αvβ6, could promote the polarization of M2 macrophages, an important profibrotic player in renal fibrosis. Thus, our study revealed IL-34 as the downstream effector of αvβ6, linking tubular αvβ6 expression with macrophage activity post-kidney injury, and thereby exacerbating renal inflammation and fibrosis. Besides, as TGF-β signaling activation was reported to contribute to macrophage infiltration recently [62], it is possible that αvβ6 regulates macrophages via activating TGF-β. Furthermore, emerging studies showed that epithelial αvβ6 can also be up-regulated in response to inflammatory cytokines from macrophages, like IL-1β [63]. Our study together with theirs provided new insight into the crosstalk between kidney tubules and macrophages [64], positioning αvβ6 as a critical target to break this feedback loop.

This study further delved into the molecular mechanism linking αvβ6 and IL-34 in renal tubular cells. Utilizing unbiased RNA sequencing, we identified that Hippo/YAP signaling was most significantly affected by *Itgb6* silencing. Subsequent experiments established that αvβ6 regulated IL-34 by directly up-regulating YAP expression and activation, a mechanism not thoroughly explored in the existing literature. Using a YAP agonist, the inhibition of IL-34 in si-*Itgb6*-transfected PTCs was reversed, restoring macrophage activity. These results elucidated that αvβ6-activated YAP signaling was involved in IL-34 overproduction, culminating in macrophage infiltration and activation. Similarly, Zheng et al. reported that YAP can recruit macrophages via increasing CCL2 expression after kidney injury to promote inflammation [65]. Therefore. our study expands the understanding of YAP and inflammation, identifying IL-34 as a new downstream effector of YAP in macrophage recruitment and pro-inflammatory differentiation. In addition to inflammation, previous researches have shown that Hippo/YAP signaling is associated with multiple pathological mechanisms, including cell-cycle arrest, proliferation, transdifferentiation, and tubulointerstitial fibrosis, which collectively contribute to CKD progression [66]. Here, we identified αvβ6 as a new upstream effector of YAP. These discoveries suggest αvβ6’s broad involvement in the CKD process by regulating YAP-related pathological activities, and may provide an innovative approach to treating YAP-related diseases.

However, our study did not further investigate the specific mechanisms by which αvβ6 regulates the Hippo/YAP pathway. Insights can be gleaned from research on other types of integrins, which have shown [67–70] that the integrin-dependent YAP activation depends on the activation of F-actin modulators, increasing YAP expression not only by inhibiting the degradation of YAP protein but also by enhancing the YAP mRNA level [8, 71–74]. It is plausible to hypothesize that αvβ6 integrin may regulate YAP through a similar integrin-dependent cytoskeletal modifications modality. In addition, previous studies have confirmed that αvβ6 participated in epithelial-mesenchymal transition (EMT), which was inherently accompanied by skeleton rearrangement [75]. Therefore, there might be parallels in how αvβ6 activates YAP, which warrants future investigation.

In summary, we identified a novel role of αvβ6 in orchestrating renal inflammation during fibrosis through the YAP/IL-34 axis. More importantly, our study provides insight into the application of targeting αvβ6 in treating kidney fibrosis, suggesting it would be a promising strategy to attenuate not only the ECM accumulation but also the inflammation.

## Materials and methods

### Animals

Wild-type C57BL/6 mice were purchased from Beijing Vital River Laboratory Animal Technology Co., Ltd. Systemic *Itgb6* knockout C57BL/6 mice were purchased from Biocytogen Pharmaceuticals (Beijing, China). PTCs-specific αvβ6 deletion and littermate control mice (*Itgb6*^*fl/fl*^*Pepck-Cre* and *Itgb6*^*fl/fl*^, C57BL/6 background) were generated using the Cre-loxp system and provided by Guangdong GemPharmatech Co., Ltd. (Guangzhou, China). The age of experimental mice was controlled at 8- to 12-week-old. All breeding and rearing facilities were completed at the Animal Experiment Center of Sun Yat-sen University of Medical Sciences. All animal experiments were conducted in strict accordance with the guidelines of the Animal Care and Use Committee of Sun Yat-sen University and approved by the Animal Ethics Committee of Sun Yat-sen University of Medical Sciences.

### Cell culture

TKPTS cells (mouse proximal tubule epithelial cell line, CRL-3361) and RAW264.7 cells (mouse macrophage cell line, TIB-71) were both obtained from ATCC and were passaged and used in the laboratory. TKPTS cells were cultured in DMEM/F-12 (Thermo Fisher Scientific, USA) containing 10% FBS, 1% streptomycin/penicillin, and 0.1% insulin (10 mg/mL). RAW264.7 cells were cultured in High Glucose DMEM (Thermo Fisher Scientific, USA) containing 10% FBS and 1% streptomycin/penicillin.

### Dataset collection

GEO database as a Gene expression profiling public platform (www.ncbi.nlm.nih.gov/geo/) was used for preliminary bioinformatics analysis of CKD patients. The inclusion criteria for the analysis were as follows: 1) the included test samples should come from both CKD patients and healthy individuals; 2) the dataset should contain at least 15 samples in each group; 3) the gene expression profiling needs to come from adult humans; 4) the original data must be provided for further exploration. According to the criteria, dataset GSE180394 was selected for analysis [11].

### Kidney specimens

Human kidney tissue sections for this study were obtained from renal biopsies of patients with CKD or normal kidney tissue of patients with renal carcinoma. CKD patients with urinary tract infections and renal tumors were excluded. Samples were obtained from the Department of Nephrology or Department of Urology, The First Affiliated Hospital of Sun Yat-sen University. All participants signed informed consent and were approved by the Ethics Committee of the First Affiliated Hospital of Sun Yat-Sen University (approval numbers 2022(602), 2016(215)).

### Kidney unilateral ischemia-reperfusion (uIRI) model

The kidney uIRI-induced renal fibrosis model was performed as described previously [76]. Briefly, male mice were anesthetized using 1% sodium pentobarbital (50 mg/kg). The left kidney was exposed through a flank incision, and ischemic injury was induced by clamping the renal pedicle with a non-traumatic microaneurysm clip (RockTech), which was removed after 45 min. During the operation, the body temperature of the mice was controlled at 37 ± 0.2 °C, and PBS was added promptly to maintain the vital signs of the mice. Sham-operated control mice underwent the same surgery without clamping the renal pedicle.

### Aristolochic acids (AA) injection-induced nephropathy (AAN) model

A model of renal fibrosis induced by AA injection as previously described [77]. Briefly, male WT and *Itgb6*^-/-^ mice were intraperitoneally injected with 5 mg/kg AA (Sigma-Aldrich, A9451) or PBS every other day, and kidneys and serum were collected 10 days later for detection. Blood urea nitrogen (BUN) and serum creatinine levels were measured by commercial reagents and biochemical analyzers (Roche).

### In vivo and in vitro treatment of recombinant mouse IL-34 (rmIL-34)

For in vivo IL-34 administration, each *Itgb6*^-/-^-uIRI mice were intraperitoneally injected with 1 μg of rmIL-34 (RD, 5195) on days 0 and 3 after uIRI. Control animals received PBS. For in vitro IL-34 treament, after hypoxia for 24 h, serial concentration gradients (0–250 ng/mL) of rmIL-34 (RD, 5195-ML) were added to the co-culture system of TKPTS cells and RAW264.7 cells, and the cells were treated for 12 h.

### Immunohistochemical staining

Renal biopsy sections from CKD patients were subjected to antigen retrieval and non-specific binding sites were blocked with 5% BSA. According to the experimental requirements, kidney sections were incubated with sheep anti-human integrin β6 antibody (Ab) (PA5-47588, Thermo Fisher Scientific), mouse anti-human CD20 Ab (ab9475, Abcam), rabbit anti-human CD3 Ab (ab5690, Abcam), or mouse anti-human CD68 Ab (ab955, Abcam) at 4 °C overnight. Primary antibodies were labeled by incubating biotin-linked secondary antibodies, respectively.

Mouse kidneys used for immunohistochemistry experiments were fixed in 4% paraformaldehyde, embedded in paraffin, and cut into 4 μm thick sections. After being blocked with 5% BSA, kidney sections were stained with rat anti-mouse F4/80 Ab (BIO-RAD, MCA497G), goat anti-mouse integrin β6 Ab (RD, AF2389), sheep anti-mouse IL-34 Ab (RD, AF5195), or rabbit anti-mouse YAP Ab (CST, 14074S) at 4 °C overnight. 3-3-diaminobenzidine (DAB) was used for color development in immunohistochemistry.

The slides were then examined on a pathological section scanner (Kfbio, KF-PRO-020). Immunohistochemistry was quantified by counting the positive areas in 10 high-power fields (HPF).

### Quantitative real-time PCR (qRT-PCR)

QRT-PCR was performed on mouse kidneys and cells. Trizol was used to lyse and extract total RNA from kidney homogenates, TKPTS cells, and RAW264.7 cells. The RNA extracted from trizol was extracted by chloroform, further precipitated in isopropanol, and washed with absolute ethanol. Finally, the RNA was dissolved in DEPC water. The concentration and quality of RNA were measured by NanoDrop-2000 spectrophotometer (Thermo Fisher Scientific, USA). The RNA was reverse transcribed into cDNA according to a commercial reverse transcription kit (Vazyme, China). A PCR system was constructed using SYBR green dye, specific primers, and cDNA, and detection was performed in Applied Biosystems 7500 (Thermo Fisher Scientific, USA). Primer sequences are shown in Table S2.

### Preparation of kidney single-cell suspension

Mice were anesthetized with 1% pentobarbital and perfused with PBS until their kidneys became pale. The kidneys were mechanically cut into chunks and minced in RPMI 1640 containing 2% FBS at low temperatures before digestion. Digestion buffers were prepared with 1 mg/mL collagenase type II (Thermo Fisher Scientific, 17101015) and 0.5 mg/mL dispase type II (Thermo Fisher Scientific, 17105041) in RPMI 1640 containing 2% FBS. The kidneys were digested in a 200 rpm oscillator at 37 °C for 30 min. Post-digestion, the digestive fluid was filtered with a 70 μm filter and centrifuged. Red blood cells were lysed by 1 ml ACK Lysis Buffer (A1049201, Thermo Fisher Scientific). Centrifugation after the termination of fission was performed and the cell pellets were resuspended with PBS to obtain single-cell suspensions of mouse kidneys.

### Flow cytometry

Single-cell suspensions from mouse kidneys were prepared, and extracellular antigens were stained with flow cytometry antibodies. The antibodies used for Flow cytometry analysis are listed in Table S3. An AttuneNxT acoustic focusing cytometer (Thermo Fisher Scientific) was used for flow cytometry analysis, and FlowJo v.10 was used to process flow cytometry results.

### Western blot

RIPA lysis buffer was used for protein extraction from mouse kidney homogenate, TKPTS cells, and RAW264.7 cells after supplementing protease inhibitors and phosphatase inhibitors. After centrifugation to remove structural proteins, the protein concentration was detected by the BCA method. Equal amounts of protein were separated by SDS-PAGE and electro-transferred to PVDF membranes. After blocking with 5% skim milk or 5% BSA, the PVDF membranes were incubated with primary antibodies overnight at 4 °C. The antibodies used in western blot were as follows: goat anti-mouse integrin β6 antibody (RD, AF2389), mouse anti-mouse α-SMA Ab (Sigma-Aldrich, A5228), rabbit anti-mouse Fibronectin Ab (BOSTER, BA1772), sheep anti-mouse IL-34 Ab (RD, AF5195), rabbit anti-mouse YAP Ab (CST, 14074 S), rabbit anti-mouse p-YAP (S127) Ab (CST, 4911 S), mouse anti-mouse GAPDH Ab (Abcam, ab8245), mouse anti-mouse α-Tubulin Ab (CST, 12351 S), and mouse anti-mouse β-actin Ab (Abcam, ab8226). After incubation was complete, unbound antibodies were washed with TBST (TBS: Tween, 1000:1). The horseradish peroxidase-conjugated secondary antibody derived from the primary antibody was incubated with the PVDF membrane for 1 h at room temperature, and the enhanced chemiluminescence (ECL) kit was used to develop specific protein bands. The image development of specific protein bands was quantitatively analyzed by ImageJ software.

### Collagen fiber detection

Kidney tissue was fixed in 4% paraformaldehyde, embedded in paraffin, and cut into 4 μm thick kidney sections. Kidney sections were stained with Sirius red dye. The severity of tubulointerstitial fibrosis was assessed by a renal pathologist who was blinded to the experimental group, and the criterion was the area of Sirius red-positive area. Scoring was performed in 10 successive HPF fields in a blinded manner.

### Immunofluorescence

Mouse kidney paraffin sections were permeabilized with 0.2% Triton X-100 after completion of antigen retrieval, and nonspecific sites were blocked with 10% donkey serum. LTL-fluorescein (Vector, FL-1321-2) was used to label proximal renal tubules, PNA-fluorescein (Vector, FL-1071-5) was used to label distal renal tubules, DBA-fluorescein (Vector, FL-1031-2) was used to label collecting ducts. Rabbit anti-mouse YAP Ab (CST, 14074 S), sheep anti-mouse IL-34 Ab (RD, AF5195), or rabbit anti-mouse KIM-1 Ab (Novus, NBP1-76701SS) was used to label the localization of YAP, IL-34, or KIM-1. The above primary antibodies were incubated overnight at 4 °C. After the unbound primary antibody was eluted with PBS, the corresponding FITC- or PE-labeled fluorescent secondary antibody was incubated for 1 h at room temperature. 4’,6-Diamidino-2-phenylindole dihydrochloride (DAPI) was used to label the cell nucleus. Confocal fluorescence microscopy (ZEISS, LSM880 with Airyscan) was used to capture fluorescent signals, and ImageJ software was used to perform quantitative statistics on the co-localization of fluorescent signals.

### Hypoxia/reoxygenation (H/R) injury cell model

In order to simulate the ischemia-reperfusion injury model in vivo, we used an in vitro H/R injury cell model. The resume of the H/R injury cell model was performed as described previously [78], but slightly adjusted according to the experimental requirements. Briefly, TKPTS cells were exposed to a hypoxic incubator (1% O_2_, 5% CO_2_, 94% N_2_, 37 °C) for 24 h, and then the cells were placed back into a normal oxygen incubator (5% CO_2_, 37 °C) for 12 h.

### RNA interference in TKPTS cells

When TKPTS cells were cultured in 6- or 12-well culture plates to a density of 60%, NEOFECT RNA transfection reagent (NEOFECT, China) was used as a carrier to transfect TKPTS cells with *Itgb6* siRNA (Gene Pharma, China). During the transfection process, the cells were placed in FBS-free Opti-MEM (Thermo Fisher Scientific, USA) for 12 h and then transferred to a complete medium as described above. The inhibition efficiency of siRNA on target genes was verified by qPCR and western blot experiments. The sequence of the *Itgb6* siRNA is shown in Table S4.

### Plasmid-transfected overexpression of *Itgb6* and blockade of IL-34 in TKPTS cells

Glycerobacteria pcDNA3.1-*Itgb6*(mouse)-3×Flag-Neo overexpressing *Itgb6* gene were inoculated into LB medium with Ampicillin double-antibody. After 37 °C and 220 rpm vibration for 12–16 h, the plasmid was extracted by alkaline lysis. When TKPTS cells were cultured in 6-well culture plates confluent to 60–80%, NEOFECT DNA transfection reagent (NEOFECT, China) was used as a vector-binding plasmid. TKPTS cells were transfected with *Itgb6* overexpression plasmid or scramble plasmid. After 8 h of transfection, the cells were observed and transferred into a complete medium. The effect of plasmid on target gene overexpression was verified by qPCR. The NCBI registration code of the *Itgb6* overexpression plasmid is NM_001159564.1. Regarding the blockade of IL-34, after hypoxia for 24 h, an anti-IL-34 antibody (αIL-34) (RD, AF5195) or isotype control (RD, 5-001-A) was added to the co-culture system of TKPTS cells and RAW264.7 cells under the concentration of 10 μg/mL, and the cells were treated for 12 h.

### Macrophage migration and differentiation assay

To further investigate the interaction between proximal tubular epithelial cells and macrophages, we constructed an in vitro co-culture system of TKPTS cells and RAW264.7 cells based on previous studies [79]. An 8 μm-pore transwell insert (Corning, USA) was used for the detection of macrophage migration, whereas a 0.4 μm-pore transwell insert (Corning, USA) was used for the detection of macrophage function. TKPTS cells were cultured in 24-well plates and treated with H/R or *Itgb6* siRNA interference according to experimental requirements. Macrophages were seeded into the upper transwell chambers at a density of 5 × 10^4^ cells per well. After co-culturing at 37 °C for 12 h, the cells in the upper compartment were gently wiped off with a sterile cotton swab. The migrating cells attached to the lower compartment were fixed with 4% paraformaldehyde for 20 min and stained with 0.1% crystal violet dye for 20 min. The chamber was washed twice with PBS, and the migration of macrophages was observed under a light microscope (Olympus, BX63) after static drying. The migratory cells were counted in six random fields of view in the transwell chamber under HPF, and the results were analyzed by ImageJ software. In the experiment of macrophage function detection, total RNA was extracted from RAW264.7 cells lysed with trizol after 12 h of co-culture at 37 °C. The molecules related to macrophage function, including *Tnf*, *Il1b*, *Ccl2*, and *Il6*, were detected by qPCR.

### ELISA

Cell culture supernatants of TKPTS cells from each experimental group were collected and the concentration of IL-34 secreted by TKPTS cells was detected using the Mouse IL-34 Elisa Kit (Elabscience, China). Experiments were performed according to the manufacturer’s protocol and OD values were measured at a wavelength of 450 nm. The IL-34 concentrations were proportional to OD450 values, and IL-34 concentrations in samples were calculated from the standards curve.

### Pharmacological activation of YAP

XMU-MP-1 is a commonly used YAP signal agonist [80]. A concentration of 2.5 μM of XMU-MP-1 (TargetMol, T4212) was added to the culture system of TKPTS cells for 6 h to activate Hippo/YAP signaling.

### RNA sequencing

The control group and experimental group were H/R injured TKPTS cells transfected with si-NC or si-*Itgb6*, respectively. Total RNA was isolated from TKPTS cells in the si-NC Group and si-*Itgb6* group (2 replicates per group) with trizol for RNA-sequencing (RNA-seq). The sequencing strategy was non-strand-specific library construction via PE150, Polya enrichment on the NOVASEQ6000 platform (GENE DENOVO, Guangzhou). Cleaned data generated by RNA-Seq were used for statistical analysis. Firstly, the cluster analysis was completed by the R language package fastcluster, and the further analysis samples were optimized. Statistics were considered to be significantly different when |fold change| > 1.2 and *p*-value < 0.05. Basic genetic diversity analysis was done with Edger. The R language package ggplot2 was used to draw the volcano mapping for visualization. The differential genes (DEGs) were analyzed by the Kyoto Encyclopedia of Genes and Genomes (KEGG) signaling pathway enrichment to explore the signal regulation mechanism of *Itgb6*.

### Quantification and statistical analysis

GraphPad Prism v9.0 software was used for data statistics and visual presentation. Experimental data were presented as mean ± SEM. An unpaired Student’s *t*-test was used to compare the two groups. In more than two groups of comparison using general one-way ANOVA for statistics. All experiments were performed in at least three biologically independent replicates. The *p*-value < 0.05 indicated a statistically significant difference. The statistical significance was respectively expressed as: *p < 0.05; **p < 0.01; ***p < 0.001; ****p < 0.0001; ^#^*p* < 0.05; ^##^*p* < 0.01; ^###^*p* < 0.001; ^####^*p* < 0.0001; ns, not significant.

### Supplementary information

Supplemental Information

Original western blots

## Data Availability

The Raw and processed transcription sequencing data of TKPTS have been deposited at the GEO with the project number: GSE253494. All other study data are included in the article and/or the supplement. Any additional data in this work are available from the corresponding authors upon request.
