# Supplementary material for: Knockout of integrin αvβ6 protects against renal inflammation in chronic kidney disease by reduction of pro-inflammatory macrophages
Source: Cell Death Dis. 2024 Jun 6;15(6):397. doi: 10.1038/s41419-024-06785-5 (PMC11156928; doi:10.1038/s41419-024-06785-5)
Supplement: Supplementary file 1 — Supplemental Information [file 41419_2024_6785_MOESM1_ESM.pdf]

Supplementary Figures and Tables: 6 figures and 4 tables

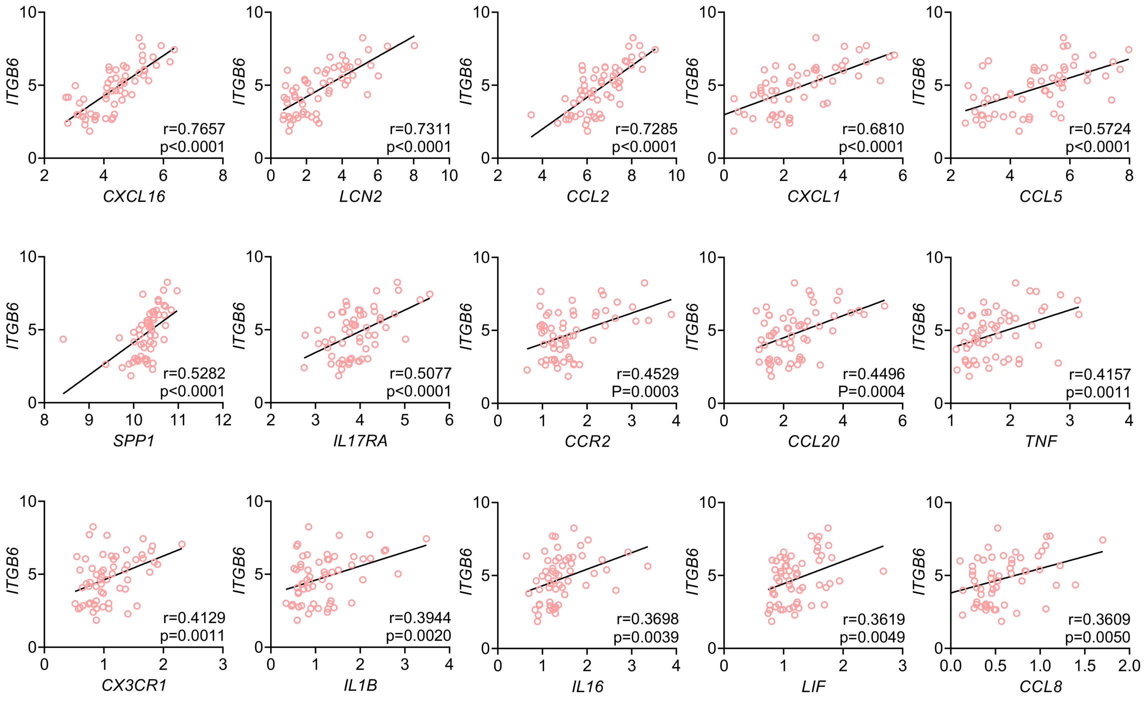

Fig. S1 Correlation analysis of the transcription expression level of *ITGB6* and inflammatory mediators in kidney specimens of healthy controls and CKD patients from dataset GSE180394. Correlation analyses were performed with Pearson correlation test (n = 59).

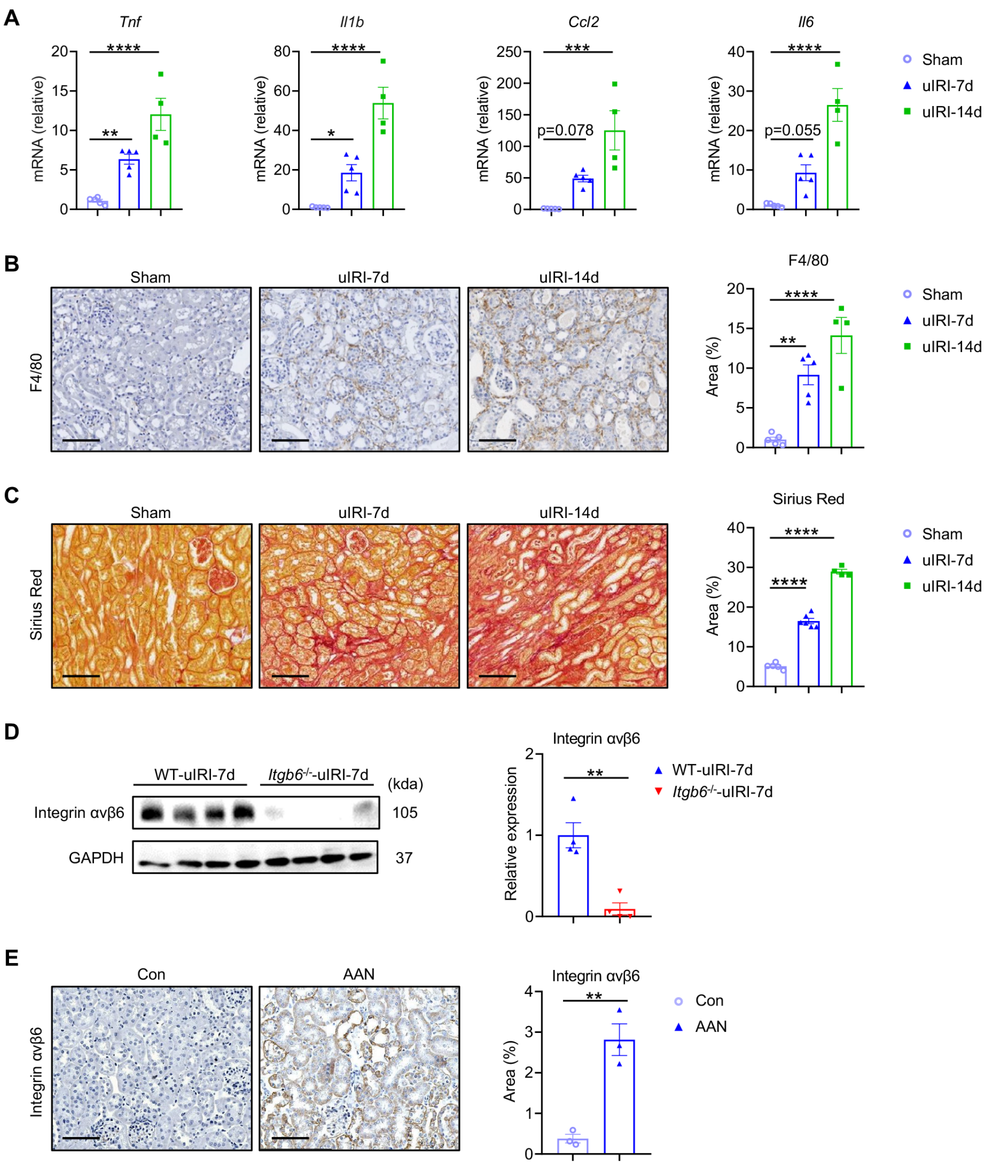

Fig. S2 IRI induces severe inflammation and fibrosis in the kidney and integrin αvβ6 deficiency attenuates renal fibrosis. A Relative mRNA levels of renal inflammatory factors (*Tnf*, *Il1b*, *Ccl2*, and *Il6*) in sham, uIRI-7d, and uIRI-14d mice were detected by qPCR (n = 4-5 per group). **B** Representative images and quantifications of F4/80 immunostaining in kidney sections of sham, uIRI-7d, and uIRI-14d mice (scale bar, 100 μm) (n = 4-5 per group). **C** Representative images and quantifications of Sirius Red staining in kidney sections from sham, uIRI-7d, and uIRI-14d mice (scale bar, 100 μm) (n = 4-5 per group). **D** Western blot of integrin αvβ6 in WT-uIRI-7d and *Itgb6*$^{-/-}$-uIRI-7d mice. The quantification of the relative levels of integrin αvβ6/GAPDH is shown (n = 4 per group). **E** Representative images and quantifications of integrin αvβ6 immunostaining in kidney sections of control and AAN mice (scale bar, 100 μm) (n = 3 per group). Data are presented as mean ± SEM of three biological replicates. One-way ANOVA (**A-C**) and Student's *t*-test (**D, E**) were performed. **p < 0.01; ****p < 0.0001.

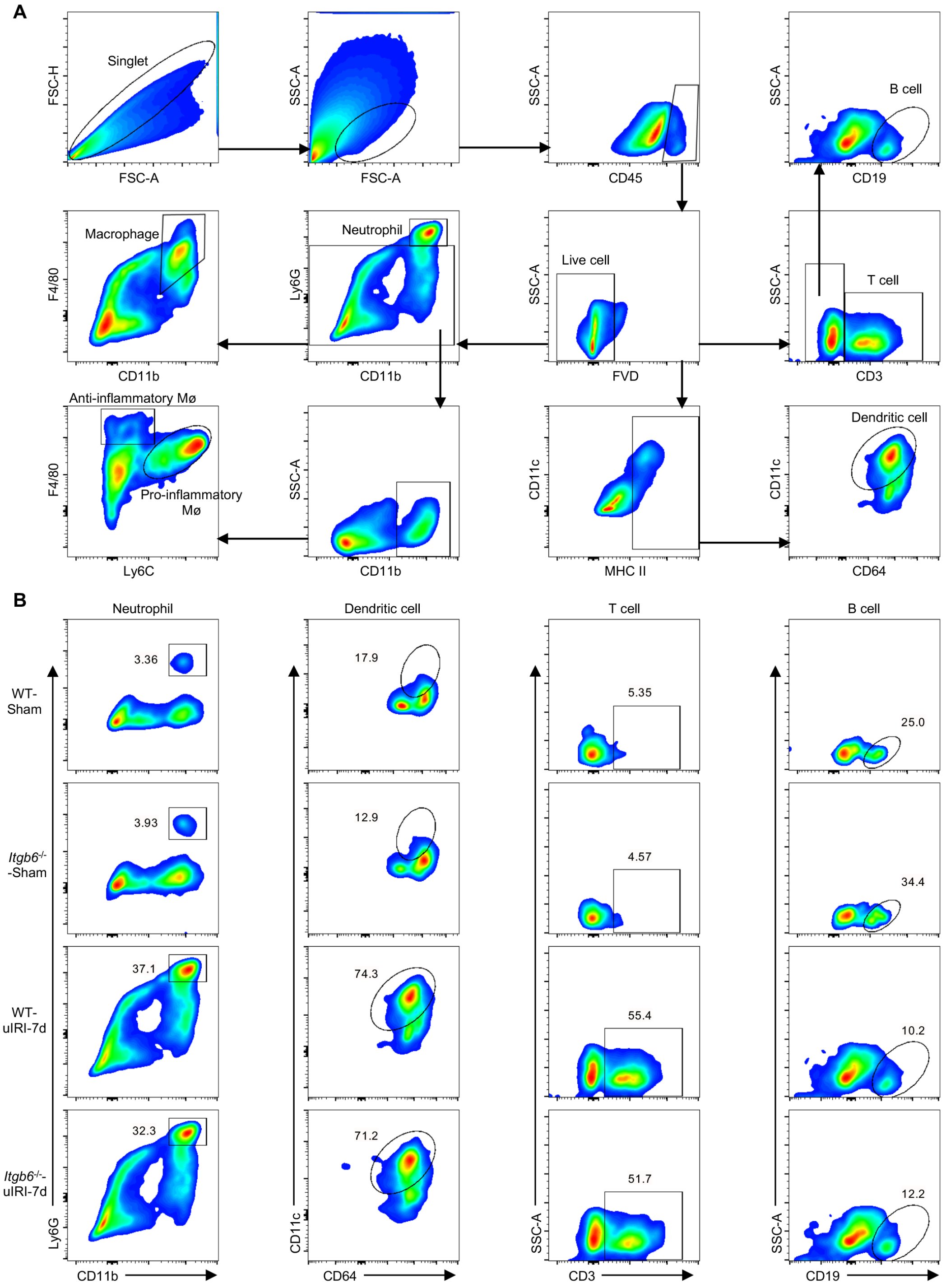

Fig. S3 Gating strategies and represented images of different immune cells in flow cytometry analyses. A Gating strategy of identifying neutrophils, dendritic cells, T cells, B cells, and macrophages, including anti-inflammatory and pro-inflammatory macrophages, in mouse kidneys. **B** Represented flow cytometric images of neutrophils, dendritic cells, T cells, and B cells in sham or uIRI-7d mice.

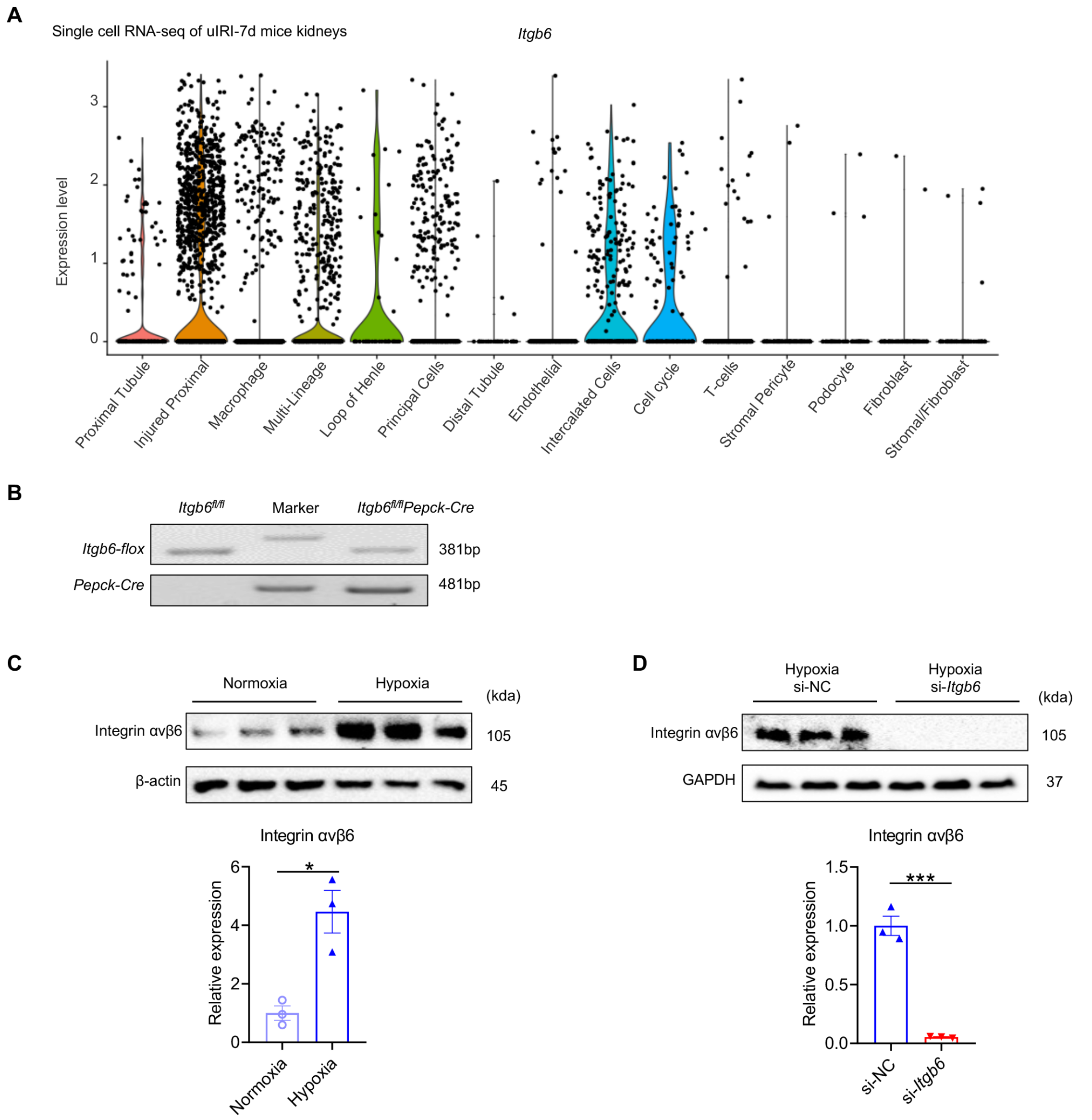

Fig. S4 Si-*Itgb6* effectively downregulates hypoxia-induced integrin αvβ6 overexpression in renal proximal tubules. A *Itgb6* expression in different cell types of uIRI-7d mice kidneys was analyzed based on a scRNA-seq dataset of GSE139506. **B** *LoxP* sites of *Itgb6* and insertion of *Pepck-cre* transgene in *Itgb6*$^{fl/fl}$ or *Itgb6*$^{fl/fl}$*Pepck-Cre* mice were confirmed by genotyping of genomic DNA from tissue lysates using PCR. **C** Western blot of integrin αvβ6 in normoxia or hypoxia-treated TKPTS cells. The quantification of the relative level of integrin αvβ6/β-actin is shown (n = 3 per group). **D** Western blot of integrin αvβ6 in si-NC or si-*Itgb6*-transfected hypoxic TKPTS cells. The quantification of the relative level of integrin αvβ6/β-actin is shown (n = 3 per group). Data are presented as mean ± SEM of three biological replicates. Student's *t*-test (**C, D**) was performed. $^*p < 0.05$; $^{***}p < 0.001$.

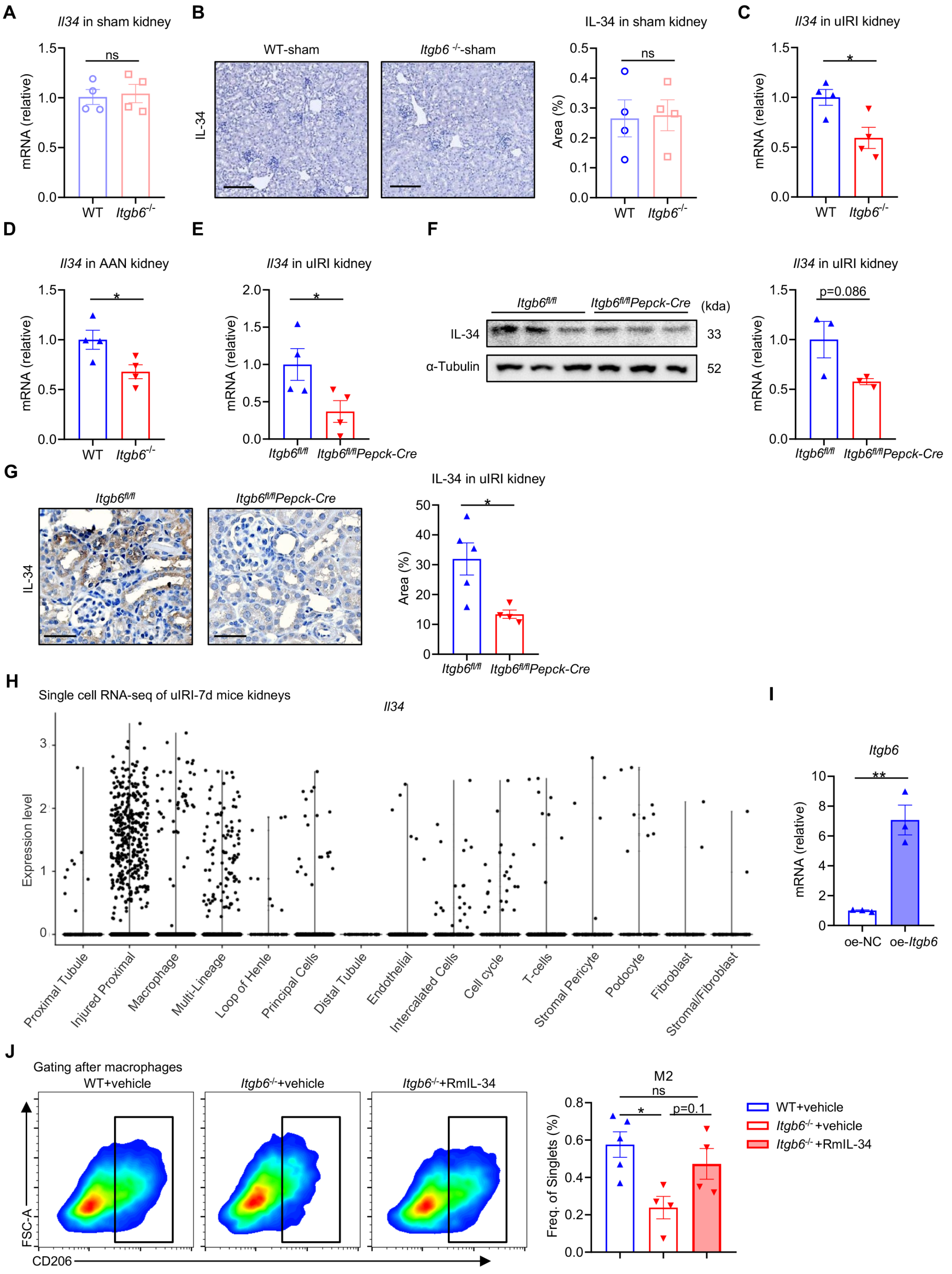

A
Il34 in sham kidney
ns
mRNA (relative)
WT
Itgb6-/-
B
WT-sham
Itgb6 -/--sham
IL-34
IL-34 in sham kidney
Area (%)
C
Il34 in uIRI kidney
*
D
Il34 in AAN kidney
E
Il34 in uIRI kidney
Itgb6fl/fl
Itgb6fl/flPepck-Cre
F
(kda)
IL-34
33
α-Tubulin
52
p=0.086
G
IL-34 in uIRI kidney
H
Single cell RNA-seq of uIRI-7d mice kidneys
Il34
Expression level
Proximal Tubule
Injured Proximal
Macrophage
Multi-Lineage
Loop of Henle
Principal Cells
Distal Tubule
Endothelial
Intercalated Cells
Cell cycle
T-cells
Stromal Pericyte
Podocyte
Fibroblast
Stromal/Fibroblast
I
Itgb6
**
oe-NC
oe-Itgb6
J
Gating after macrophages
WT+vehicle
Itgb6-/-+vehicle
Itgb6-/-+RmIL-34
FSC-A
CD206
M2
p=0.1
Freq. of Singlets (%)

Fig. S5 *Itgb6* knockout downregulates renal IL-34 expression in uIRI but not sham kidney. **A** Relative mRNA levels of *Il34* in kidneys of WT-sham or *Itgb6*$^{-/-}$-sham mice were detected by qPCR (n = 4 per group). **B** Representative images and quantifications of IL-34 immunostaining in kidney sections from WT-sham or *Itgb6*$^{-/-}$-sham mice (scale bar, 100 μm) (n = 4 per group). **C, D** Relative mRNA levels of *Il34* in uIRI-7d (**C**) or AAN (**D**) kidneys of WT and *Itgb6*$^{-/-}$ mice were detected by qPCR (n = 3-4 per group). **E** Relative mRNA levels of *Il34* in uIRI-7d kidneys of *Itgb6*$^{fl/fl}$ or *Itgb6*$^{fl/fl}$*Pepck-Cre* mice were detected by qPCR (n = 4-5 per group). **F, G** Western blot and immunostaining of IL-34 in uIRI-7d kidneys of *Itgb6*$^{fl/fl}$ or *Itgb6*$^{fl/fl}$*Pepck-Cre* mice (scale bar, 50 μm). The quantification of the relative levels of IL-34/α-tubulin and quantification of IL-34 positive area proportion are shown (n = 4-5 per group). **H** Expression of *Il34* in different cell types of uIRI-7d mice kidneys was analyzed based on a scRNA-seq dataset of GSE139506. **I** Relative mRNA levels of *Itgb6* in oe-NC or oe-*Itgb6*-transfected hypoxic TKPTS cells were detected by qPCR (n = 3 per group). **J** Comparison of M2 macrophages (CD206^{+}) in WT and *Itgb6*$^{-/-}$ mice supplemented with rmIL-34 or vehicle (n = 4-5 per group). Data are presented as mean ± SEM of three biological replicates. Student's *t*-test (**A-H**) and one-way ANOVA (**J**) were performed. *p < 0.05; **p < 0.01; ns, not significant.

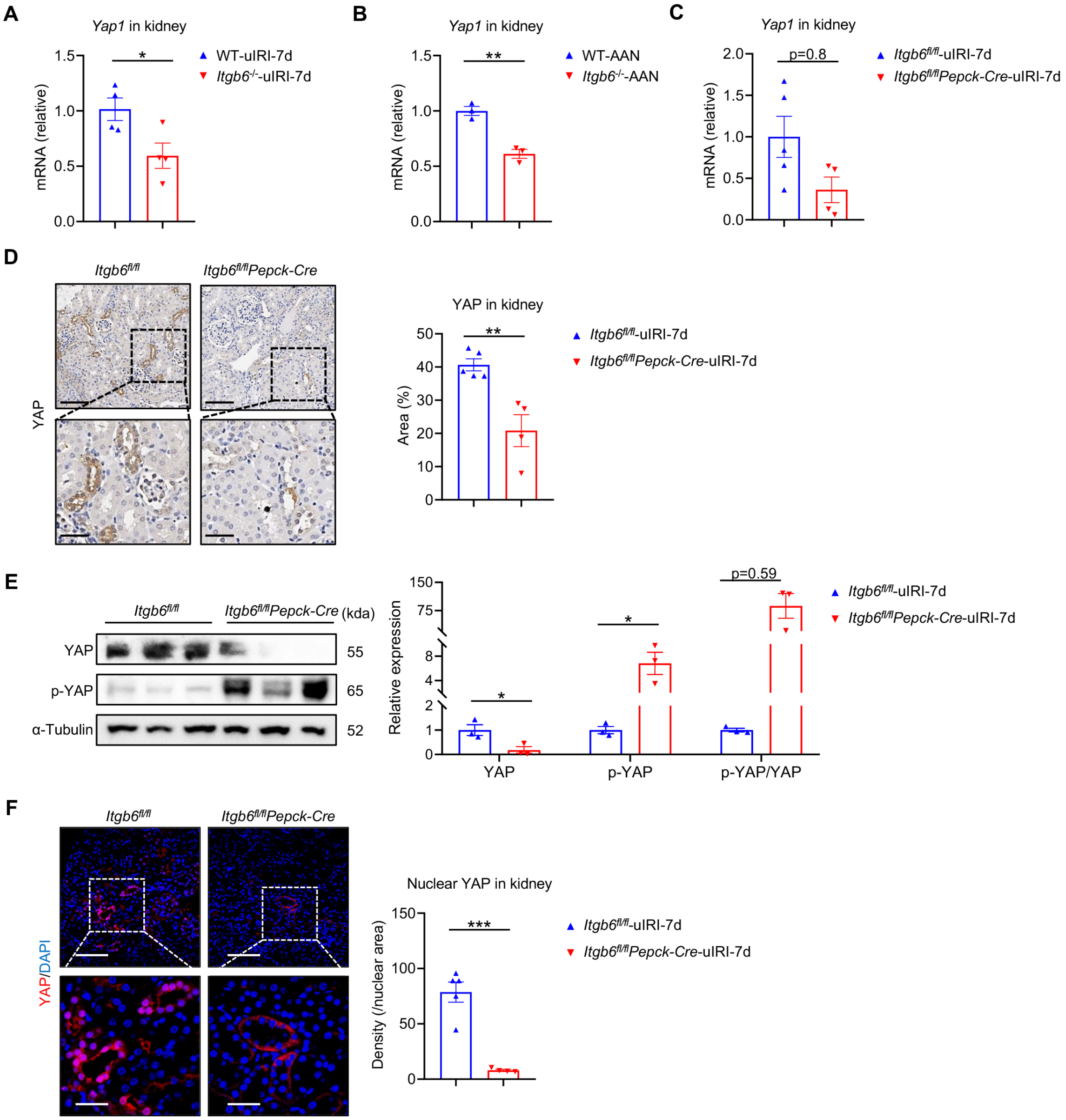
A
Yap1 in kidney
mRNA (relative)
WT-uIRI-7d
Itgb6-/--uIRI-7d
B
Yap1 in kidney
WT-AAN
Itgb6-/--AAN
C
Yap1 in kidney
p=0.8
Itgb6fl/fl-uIRI-7d
Itgb6fl/flPepck-Cre-uIRI-7d
D
Itgb6fl/fl
Itgb6fl/flPepck-Cre
YAP
YAP in kidney
Area (%)
E
(kda)
YAP
p-YAP
α-Tubulin
55
65
52
Relative expression
p=0.59
p-YAP/YAP
F
YAP/DAPI
Nuclear YAP in kidney
Density (/nuclear area)

Fig. S6 *Itgb6* knockout decreases the mRNA levels of *Yap1* in uIRI-7d mice.

A, B Relative mRNA levels of *Yap1* in uIRI-7d (**A**) or AAN (**B**) kidneys of WT or *Itgb6*$^{-/-}$ mice were detected by qPCR (n = 3-4 per group). **C** Relative mRNA levels of *Yap1* in uIRI-7d kidneys of *Itgb6*$^{fl/fl}$ or *Itgb6*$^{fl/fl}$*Pepck-Cre* mice were detected by qPCR (n = 3-4 per group). **D** Immunostaining of YAP in uIRI-7d kidneys from *Itgb6*$^{fl/fl}$ or *Itgb6*$^{fl/fl}$*Pepck-Cre* mice. The quantification of YAP positive area proportion is shown (scale bar, 100 μm). The boxed area in the upper panels is magnified in the lower panels (scale bar, 50 μm) (n = 4-5 per group). **E** Western blot of YAP and Ser127-phosphorylated YAP (p-YAP) in uIRI-7d kidneys from *Itgb6*$^{fl/fl}$ or *Itgb6*$^{fl/fl}$*Pepck-Cre* mice mice. The quantifications of the relative levels of YAP/α-tubulin or p-YAP/α-tubulin and p-YAP/YAP are shown (n = 3 per group). **F** Representative images of immunofluorescent staining for YAP (red) and DAPI (blue) in uIRI-7d kidneys from *Itgb6*$^{fl/fl}$ or *Itgb6*$^{fl/fl}$*Pepck-Cre* mice. Semi-quantification of nuclear YAP expression is shown (Scale bar, 50 μm). The boxed area in the upper panels is magnified in the lower panels (scale bar, 25 μm) (n = 4-5 per group). Data are presented as mean ± SEM of three biological replicates. Student's *t*-test was performed. *$p < 0.05$; **$p < 0.01$; ***$p < 0.001$.

Table S1. Clinical characteristics of healthy controls and CKD patients for detecting renal integrin αvβ6, B cell, T cell and macrophage by immunocytochemistry staining.

Group	Gender (M/F)	Age in years	Serum creatinine in µmol/L
Healthy controls	2/1	44.0 ± 8.7	64.7 ± 14.0
CKD patients	5/1	31.7 ± 9.8	156.7 ± 62.0

Data are presented as n or mean ± standard deviations. M: male: F: female.

Table S2. Primers for quantitative real-time PCR analysis.

Gene name	Primer sequence forward (5′ ➔ 3′)	Primer sequence reverse (5′ ➔ 3′)
Actb	ACCCGCGAGCACAGCTTCTTTG	ACATGCCGGAGCCGTTGTCGAC
Tnf	CTGAACTTCGGGGTGATCGG	GGCTTGTCACTCGAATTTTGAGA
Il1b	TCGTGAATGAGCAGACAG	AGAGGCAAGGAGGAAAAC
Ccl2	GGCTCAGCCAGATGCAGTTAA	GTGAATGAGTAGCAGCAGGTGAGT
Il6	GCTACCAAACTGGATATAATCAGGA	CCAGGTAGCTATGGTACTCCAGAA
Csf1	GTGTCAGAACACTGTAGCCAC	TCAAAGGCAATCTGGCATGAAG
Csf2	GGCCTTGGAAGCATGTAGAGG	GGAGAACTCGTTAGAGACGACTT
Hmgb1	GGGAGGAGCACAAGAAGAAGCA	GGGCGGTACTCAGAACAGAACAAG
Mif	TCGTGAATGAGCAGACAG	AGAGGCAAGGAGGAAAAC

Cxcl2	GAAGTCATAGCCACTCTCAAGG	CCTCCTTTCCAGGTCAGTTAGC
Ccl1	GCTTACGGTCTCCAATAGCTGC	GCTTTCTCTACCTTTGTTCAGCC
Ccl3	GCCCTTGCTGTTCTTCTCTGT	GGCATTCAGTTCCAGGTCAGT
Ccl5	GACACCACTCCCTGCTGCTT	ACAAACACGACTGCAAGATTGG
Ccl7	CCACATGCTGCTATGTCAAGA	ACACCGACTACTGGTGATCCT
Ccl19	TGTGGCCTGCCTCAGATTAT	TTCCGCATCATTAGCACCCC
Ccl20	CCAGGCAGAAGCAAGCAACT	TCGGCCATCTGTCTTGTGAA
Il34	TTGCTGTAAACAAAGCCCCAT	CCGAGACAAAGGGTACACATTT
Mst1	CTCACCACTGAATGACTTCCAG	AAGGCCCGACAGTCCAGAA
Mst2	CGGGGTCCGTTTCAGACATAA	GCGTTTTGCCATTGTATCTGTT
Lats1	AAAGCCAGAAGGGTACAGACA	CCTCAGGGATTCTCGGATCTC
Lats2	GGACCCCAGGAATGAGCAG	CCCTCGTAGTTTGCACCACC
Yap1	TTTCGGCAGGCAATACGGA	CTGCTCCAGTGTAGGCAACT
Taz	CATGGCGGAAAAAGATCCTCC	GTCGGTCACGTCATAGGACTG
Tead1	GAGCGACTCGGCAGATAAGC	CCACACGGCGGATAGATAGC
Tead2	GAAGACGAGAACGCGAAAGC	GATGAGCTGTGCCGAAGACA
Tead3	CAACCAGCACAATAGCGTCCA	CTGAAAGCTCTGCTCGATGTC
Tead4	ACAATGATGCAGAGGGTGTATG	TCCTCCGTCAGGATAATTTTGC

Table S3. Fluorochrome-conjugated antibodies for flow cytometry.

Antibody	Source	Identifier
Anti-mouse CD45-EF450 Ab	EBioscience	Cat: #48-0451-82
Anti-mouse FVD-EF780 Ab	EBioscience	Cat: #65-0865-18
Anti-mouse CD11b-SB600 Ab	EBioscience	Cat: #63-0112-82
Anti-mouse Ly6G-FITC Ab	Biolegend	Cat: #127606
Anti-mouse F4/80-APC Ab	EBioscience	Cat: #17-4801-82
Anti-mouse Ly6C-PE/Cyanine7 Ab	Biolegend	Cat: #108416
Anti-mouse CD45-PE Ab	Biolegend	Cat: #110707
Anti-mouse CD11c-PE/Cyanine7 Ab	Biolegend	Cat: #117317
Anti-mouse MHC II-PE/Cyanine5.5 Ab	EBioscience	Cat: #36-5321-85
Anti-mouse CD64-BV421 Ab	Biolegend	Cat: #139309
Anti-mouse CD45-APC750 Ab	Biolegend	Cat: #103154
Anti-mouse FVD-EF506 Ab	EBioscience	Cat: #65-0866-14
Anti-mouse CD3- AF700 Ab	Biolegend	Cat: #100216
Anti-mouse CD19-Pacific Blue Ab	Biolegend	Cat: #115523
Anti-mouse CD206-FITC Ab	Biolegend	Cat: #141704

Ab: antibody.

Table S4. RNA sequences for interference with *Itgb6* in TKPTS cells.

Target gene	Sequence forward (5′ ➔ 3′)	Sequence reverse (5′ ➔ 3′)
Si-*Itgb6*	GCAACUUUAGACUGGGCUUT T	AAGCCCAGUCUAAAGUUGCT T
