# Supplementary material for: Knockout of integrin αvβ6 protects against renal inflammation in chronic kidney disease by reduction of pro-inflammatory macrophages
Source: Cell Death Dis. 2024 Jun 6;15(6):397. doi: 10.1038/s41419-024-06785-5 (PMC11156928; doi:10.1038/s41419-024-06785-5)

Fig 2

G

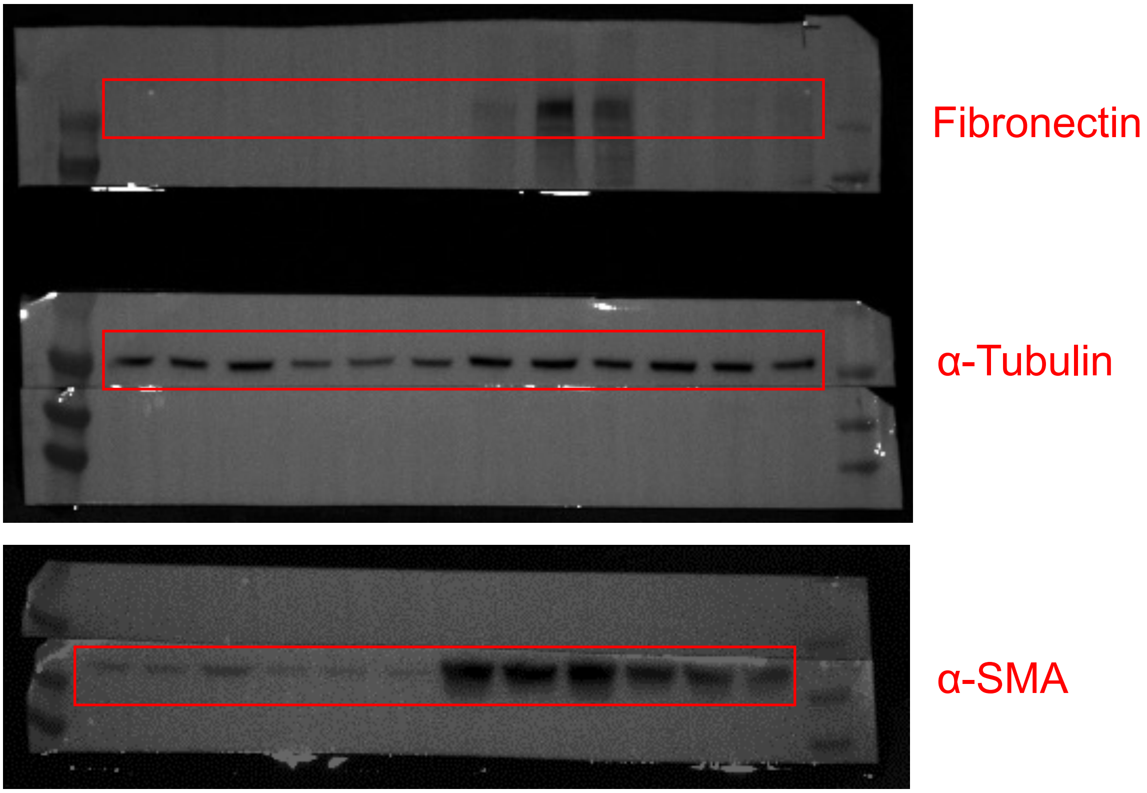

L

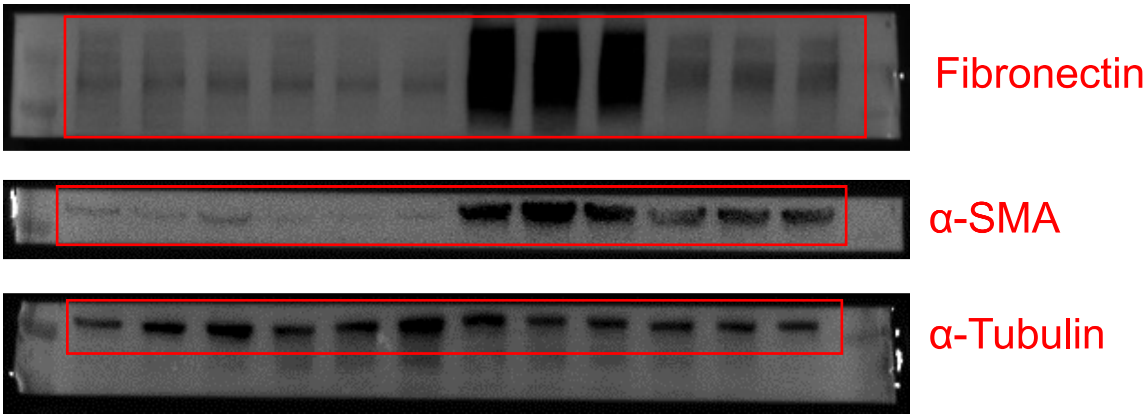

Fig 3

F

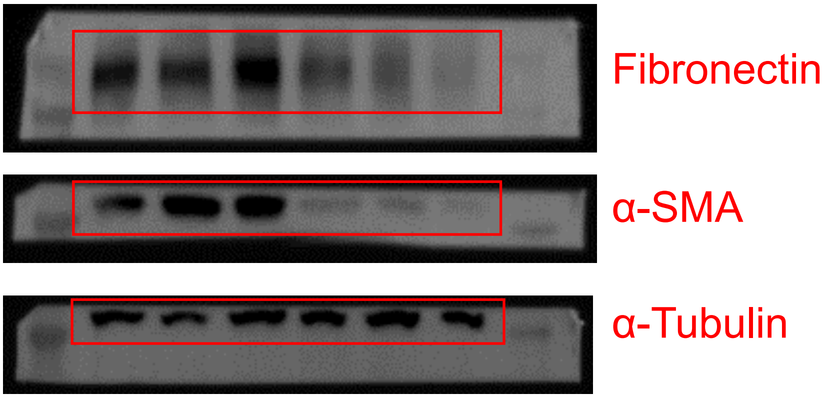

Fig 4

B

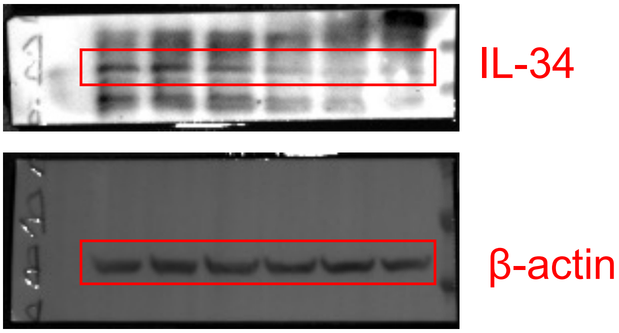
IL-34
β-actin

D

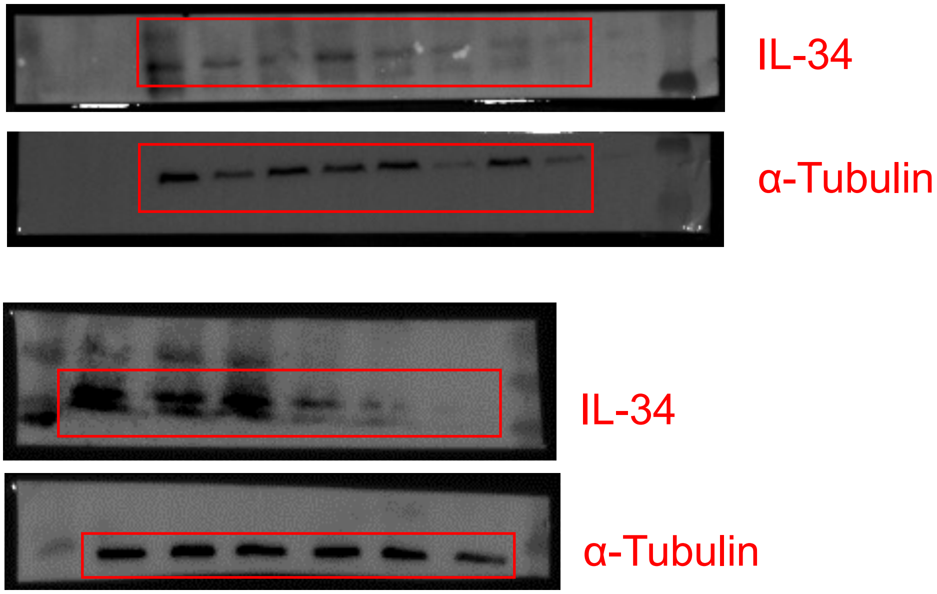
IL-34
α-Tubulin
IL-34
α-Tubulin

P

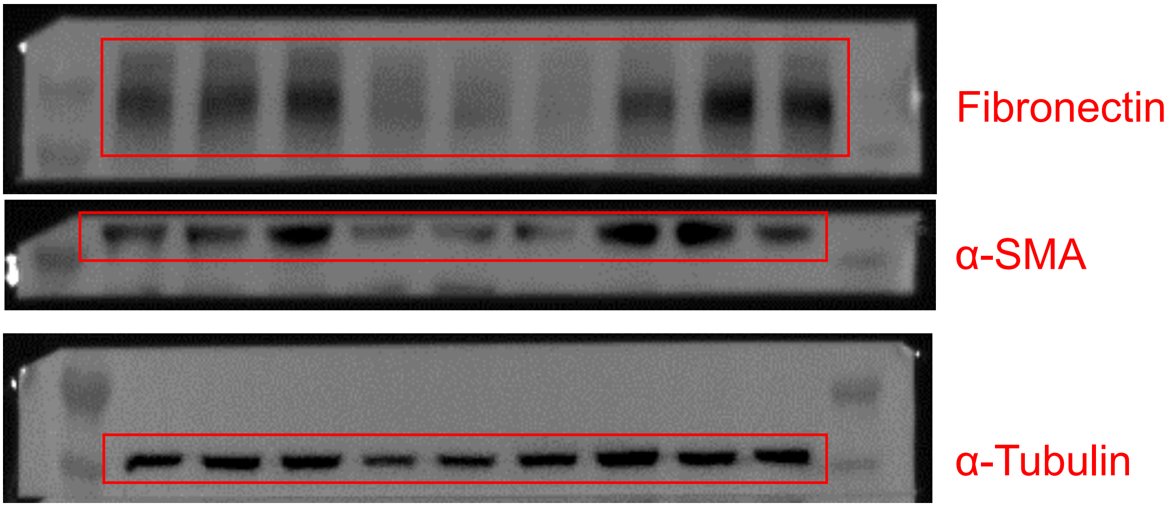
Fibronectin
α-SMA
α-Tubulin

Fig 5

D

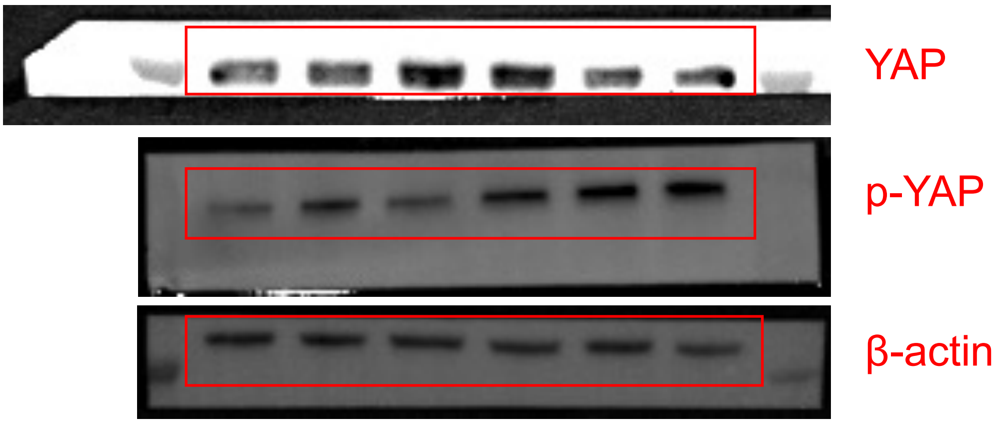

H

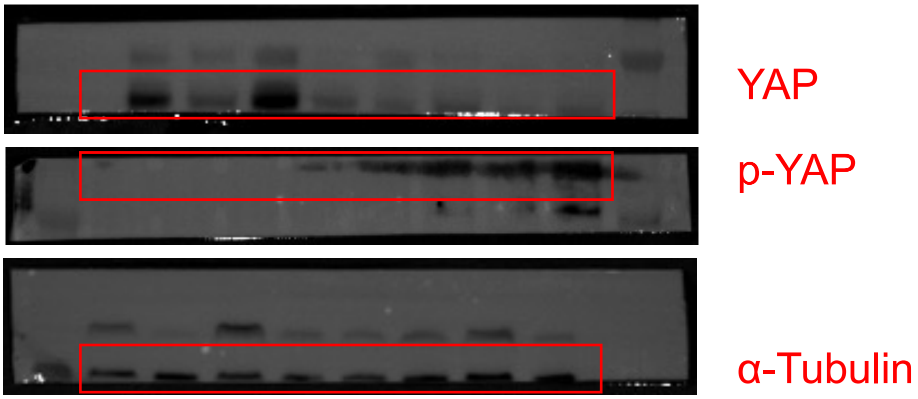

H (Reverse vision)

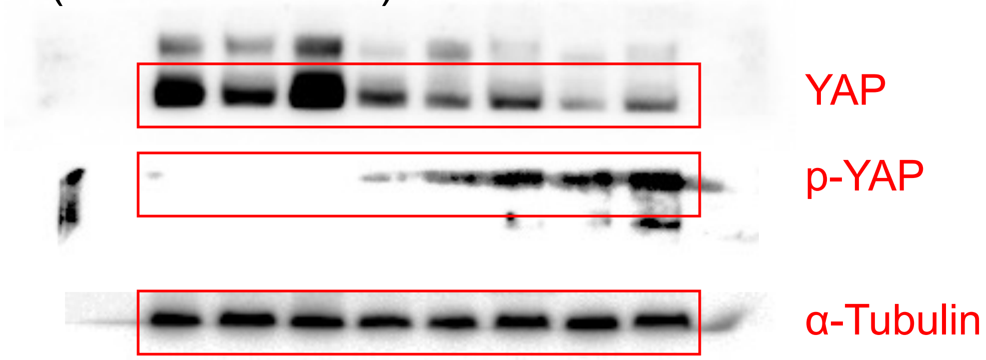

I

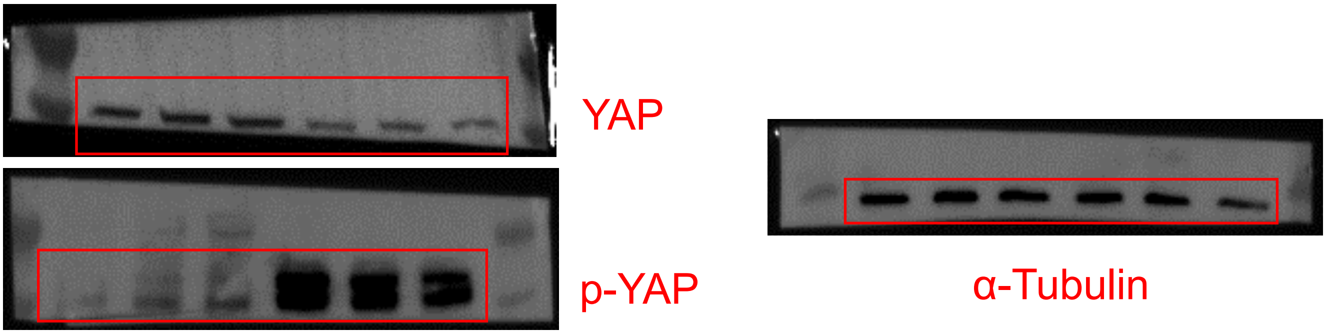

Fig 6

C

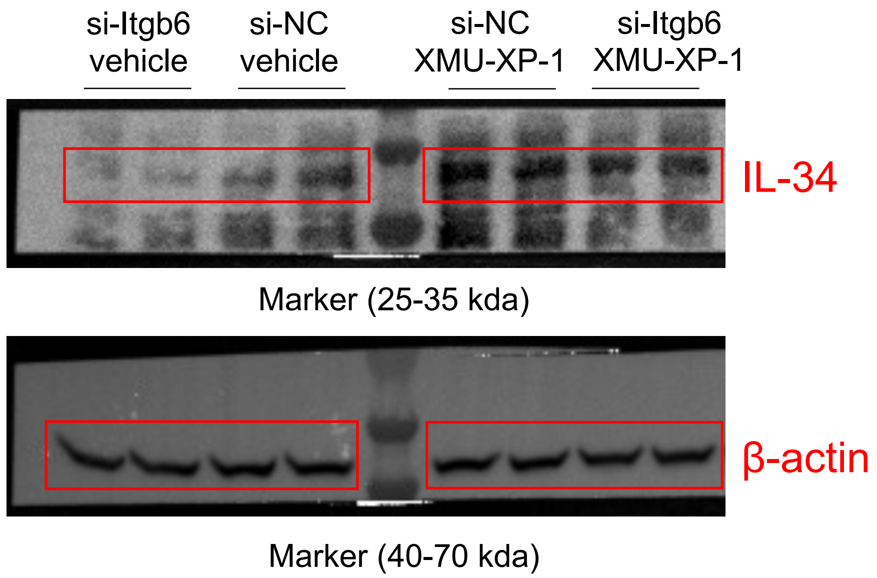
si-Itgb6
vehicle
si-NC
vehicle
si-NC
XMU-XP-1
si-Itgb6
XMU-XP-1
IL-34
Marker (25-35 kda)
β-actin
Marker (40-70 kda)

Fig. S2

D

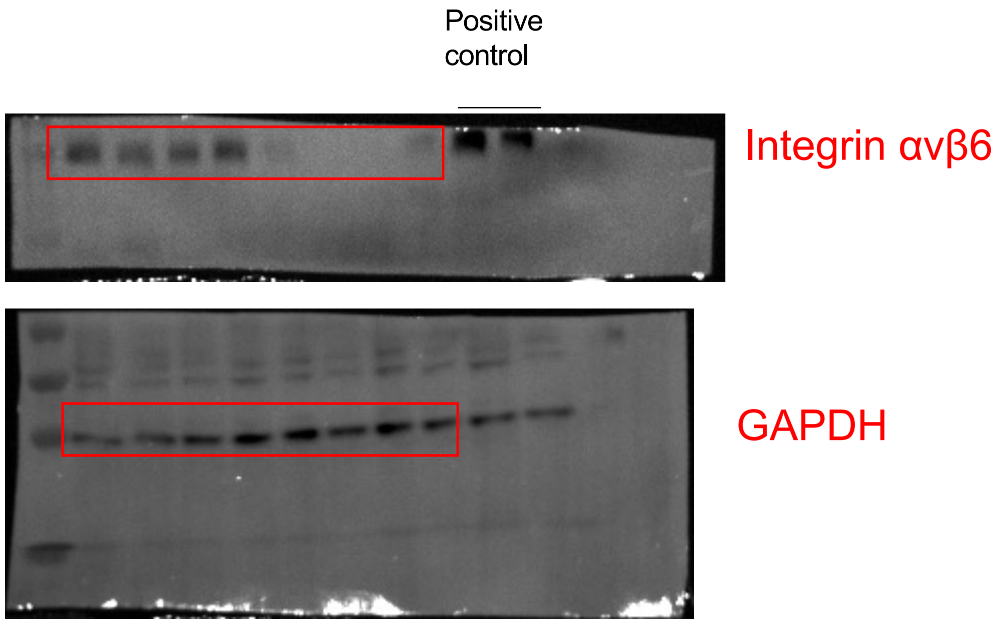

Fig S4

C

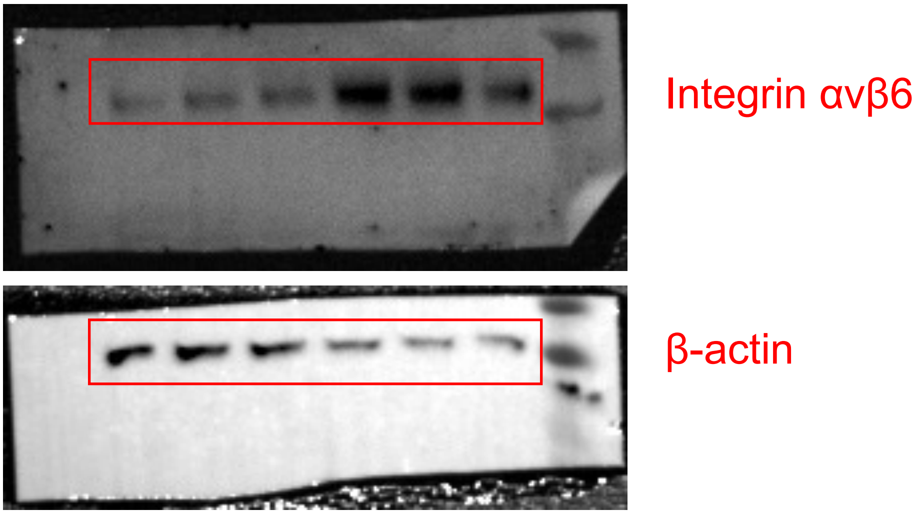

D

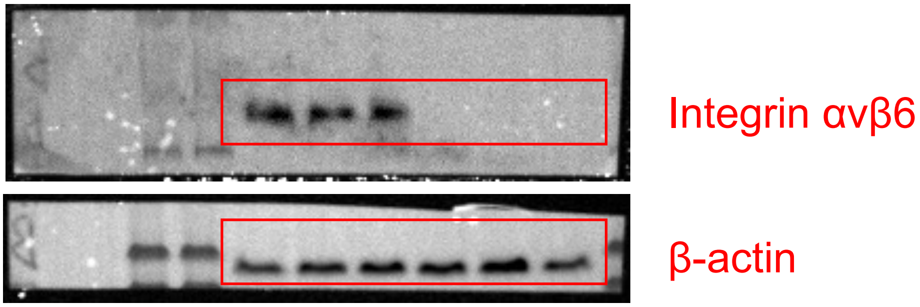

Fig S5

F

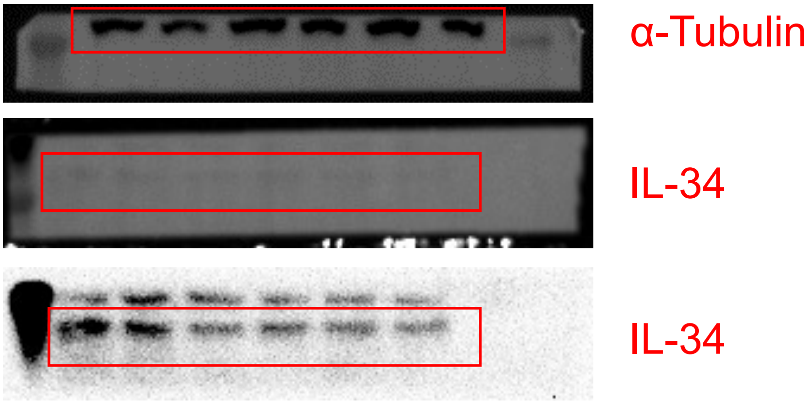

Fig S6

E

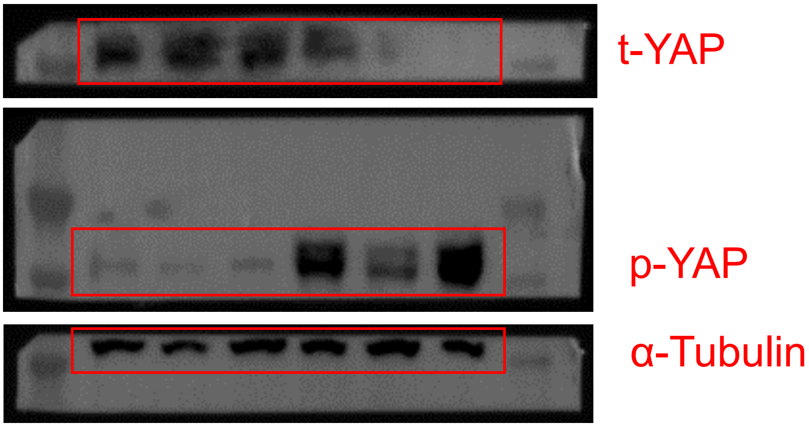

Supplement: Supplementary file 2 — Original western blots [file 41419_2024_6785_MOESM2_ESM.pdf]
